# The impact of bright light therapy on non-motor symptoms in patients with Parkinson’s disease: a systematic review and meta-analysis

**DOI:** 10.3389/fneur.2026.1770673

**Published:** 2026-03-03

**Authors:** Junjie Geng, Han li Yu, Liping Tan

**Affiliations:** 1 School of Nursing, Suzhou Medical College, Soochow University, Suzhou, Jiangsu, China; 2 Department of Nursing, The Second Affiliated Hospital of Soochow University, Suzhou, Jiangsu, China

**Keywords:** bright light therapy, meta-analysis, non-motor symptoms, Parkinson’s disease, sleep

## Abstract

**Background:**

The non-motor symptoms of Parkinson’s patients seriously affect their quality of life. This meta-analysis intended to systematically examine the beneficial effect of bright light therapy (BLT) on non-motor symptoms in individuals with Parkinson’s disease (PD).

**Methods:**

A systematic search was conducted in PubMed, Cochrane Library, Web of Science, Embase, Ovid Medline, China National Knowledge Infrastructure (CNKI), WanFang VIP and CBM to comprehensively collect RCTS and NRCTS relevant to BLT for Parkinson’s disease. Data extraction and literature screening were carried out separately by two researchers, and used the RoB2 tool and ROBINS-I tool to evaluate the risk of bias for the two types of studies, respectively. Software such as RevMan5.4 and Stata 18.0 were used to analyze the data.

**Results:**

Compared with dim light, BLT has a considerable benefit in improving the nighttime sleep of people with Parkinson’s disease. However, its therapeutic effects on depression, anxiety, exhaustion, cognition, sleep quality and quality of life are not superior to those of dim light. Moreover, the distance of the light source will alter the therapeutic effect of BLT on nighttime sleep and daytime sleepiness.

**Results:**

Compared with dim light, BLT has a considerable benefit in improving the nighttime sleep of people with PD. However, its therapeutic effects on depression, anxiety, fatigue, cognition, sleep quality and quality of life are not superior to those of dim light. Moreover, the distance of the light source will alter the therapeutic effect of BLT on nighttime sleep and daytime sleepiness.

**Conclusion:**

BLT can be used to treat the non-motor symptoms of PD and may become a practical option for home self-management. However, the ideal therapy parameters (such as intensity, distance, cycle, etc.) still need further research.

**Systematic review registration:**

https://www.crd.york.ac.uk/PROSPERO/view/CRD420251047102, identifier (CRD420251047102).

## 1 Introduction

Parkinson’s disease (PD) is a neurological disorder that usually affects middle-aged and elderly adults. It is estimated that the number of PD patients worldwide may reach 12.9 million by 2040, making PD a major global public health issue (1). Besides the motor symptoms such as resting tremors and bradykinesia, PD patients may also suffer from various non-motor symptoms such as mood disorders, speech disorders, sleep disorders and fatigue, and these non-motor symptoms can occur at any stage of the disease (2). The incidence of non-motor symptoms is 97.3–100%, and each PD patient has at least one non-motor symptom (2). The relevant research findings show (3) that 70.2% of the PD patients participating in the survey have a high severity of non-motor symptoms, and patients with a heavy burden of non-motor symptoms often have a poorer quality of life in daily life (4). Moreover, non-motor symptoms do not exist independently; one symptom can trigger or aggravate other symptoms (5), and multiple symptoms increase the psychological burden of patients and affect their social activities and normal work (6). Therefore, the pain and distress caused by non-motor symptoms to PD patients far exceed those of motor symptoms (5, 7–10). How to effectively treat and alleviate the non-motor symptoms of PD patients has become a key and core task for improving the overall quality of life of patients and reducing the burden of the disease throughout the cycle.

Drug treatment is the primary approach for managing the non-motor symptoms of PD patients. Although it can effectively alleviate the severity of symptoms, long-term use may worsen motor symptoms, disrupt autonomic nerve function, and cause mental and cognitive problems even if it can successfully reduce the intensity of symptoms (7, 11). Non-pharmacological treatments can also alleviate the non-motor symptoms of PD patients, but they still have some limitations: cognitive behavioral therapy can also help PD patients with their mood and sleep issues, but patient compliance is frequently low, and it is not appropriate for PD patients with cognitive impairments and speech disorders (7); the effectiveness of repetitive transcranial magnetic stimulation in improving patients’ emotional symptoms is still up for debate (7); and exercise therapy raises the risk of falls among patients (12). Therefore, neither drug nor non-pharmacological treatments have been able to fully balance efficacy, safety, and universality. Clinical personnel still need to seek safer and more efficient treatment approaches.

Bright light therapy (BLT) is easy to administer, noninvasive and suitable for use at home. It exerts therapeutic benefits by altering circadian rhythms and brain activity and has shown promise therapeutic effects in a range of disease groups (13–15). Currently, BLT has been used to treat non-motor symptoms of PD patients, but the results of various studies are inconsistent (16–18). A meta-analysis showed that BLT did not significantly improve anxiety, fatigue, or quality of life in PD patients, but the evidence base for its conclusions was weak due to the small number of included studies (17); the current meta-analysis only included randomized controlled trials (RCTs) (16, 17), ignoring the real-world efficacy data, which may limit the promotion of the home and routine medical settings. Moreover, the sample size of the included analysis in the meta-analysis was limited, limiting the statistical power of the results and the stability of the conclusion. This study aims to integrate the evidence from non-randomized controlled trials (NRCTs) to increase the sample size and improve the interpretability of the results. It also hopes to provide more evidence-based evidence that is closer to actual clinical practice in order to define the applicable circumstances and clarify the clinical application value of this therapy.

## 2 Methods

The meta-analysis was conducted following the PRISMA guidelines (19). The protocol was recorded on PROSPERO with the registration number CRD420251047102.

### 2.1 Search strategy

Nine databases were searched. PubMed, Cochrane Library, Web of Science, RCTs and NRCTs were systematically retrieved from Embase, Ovid Medline, China Knowledge Network Database (CNKI), Wan fang The Chinese Science and Technology Journal Database (VIP) and the Chinese Biomedical Database (CBM). The search period spans the period from the foundation of the database to September 8, 2025. After reading the title, abstract, and full text, two researchers screened the literature, independently reviewed any differences, and, if needed, sought advice from a third researcher.

### 2.2 Inclusion criteria

Inclusion in the study will be based on the following criteria: (1) All patients who meet the diagnostic criteria for Parkinson’s disease are receiving stable medication treatment; (2) No restrictions are placed on the patients’ Hoehn-Yahr stage; (3) BLT is the intervention measure for the experimental group, with no limitations on the intensity of light or the intervention environment; (4) The study type is either a RCT or a NRCT; (5) At least one indicator of interest is present, such as sleep, cognition, depression, anxiety, quality of life, and fatigue.

### 2.3 Exclusion criteria

Studies will be excluded if they meet the following criteria: (1) not Chinese-English literature; (2) review, meta-analyses, commentaries, dissertations, abstracts of academic conferences or animal experiments; (3) full text or data cannot be obtained; (4) literature that has been published multiple times with the same or incomplete information; (5) the intervention measures of the experimental group included other treatment methods; (6) case–control study.

### 2.4 Outcome indicators

This study mainly focused on the outcomes of BLT for non-motor symptoms inpatients with PD. The following are the main outcome measures: (1) depression: Beck Depression Inventory (BDI), Hamilton Depression Rating Scale (HDRS), etc. (2) Anxiety: Hamilton Anxiety Rating Scale (HAMA), etc. (3) Cognition: Mini-Mental State Examination (MMSE) and Montreal Cognitive Assessment (MoCA). (4) Fatigue: Fatigue Impact Scale (FIS), Fatigue Severity Scale (FSS), etc. (5) Daytime sleepiness: Epworth Sleepiness Scale (ESS), etc.; (6) Nighttime sleepiness: Parkinson’s Disease Sleep Scale (PDSS) and RBD questionnaire-Hong Kong (RBDQ-HK), etc.: It mainly refers to the sleep disorders experienced by patients with PD, including: the ability to maintain sleep during the night, the impact of motor symptoms on sleep, and symptoms of rapid eye movement sleep behavior disorder, etc. (7) sleep quality: Pittsburgh Sleep Quality Index (PSQI), etc.: The patient makes a subjective assessment of the quality, efficiency, duration and impact on daytime activities of their own sleep.

The secondary outcome measures were quality of life, Parkinson’s Disease Questionnaire (39-item) (PDQ-39), Unified Parkinson’s Disease Rating Scale (UPDRSII), etc.

### 2.5 Data extraction

Two researchers independently read the retrieved literature and extracted the following information from the included studies: author, publication year, region, patient characteristics, intervention content of the experimental and control groups, intervention duration, frequency, cycle, distance from the light source, and instruments.

### 2.6 Quality evaluation methods

The risk of bias in the included studies was evaluated separately by two researchers. The Cochrane RoB tool (20), which covers random sequence generation, deviation from intended interventions, missing outcome data, outcome assessment, and selective result reporting, was used to assess the quality of RCTs. Each domain was classified as “low risk,” “some concerns,” or “high risk.” For non-randomized controlled trials, the risk of bias was evaluated using ROBINS-I V2 (21), including confounding bias, intervention classification bias, selection bias, deviation from intended interventions, missing data bias, outcome measurement bias, and selective reporting of results. “low risk,” “medium risk,” “high risk,” and “extremely high risk” were the four levels (from low to high) into which each domain was divided.

### 2.7 Data analysis

Data analysis was performed using RevMan 5.4 and Stata 18.0. Standardized mean differences (SMD) with 95% confidence intervals (CI) were used to express the effect sizes between the experimental and control groups for continuous variables. When multiple scales assessed the same outcome, the mean was multiplied by −1 (indicating that higher scores reflected more severe symptoms) (22). In the heterogeneity test, Cochran’s Q test (with significance level a = 0.10) and the I^2^ statistic were utilized combined for assessment. The I^2^ value was used to measure the degree of heterogeneity, and the random effects model was used in the meta-analysis when I^2^ ≥ 50%. If *p* < 0.10, it was considered that there was significant statistical heterogeneity among the studies (23). Employ Egger’s test to evaluate publication bias. Sensitivity study evaluated the robustness of aggregated findings. The level needed for statistical significance was established at *p* < 0.05.

## 3 Results

### 3.1 Literature retrieval

**Figure 1:**
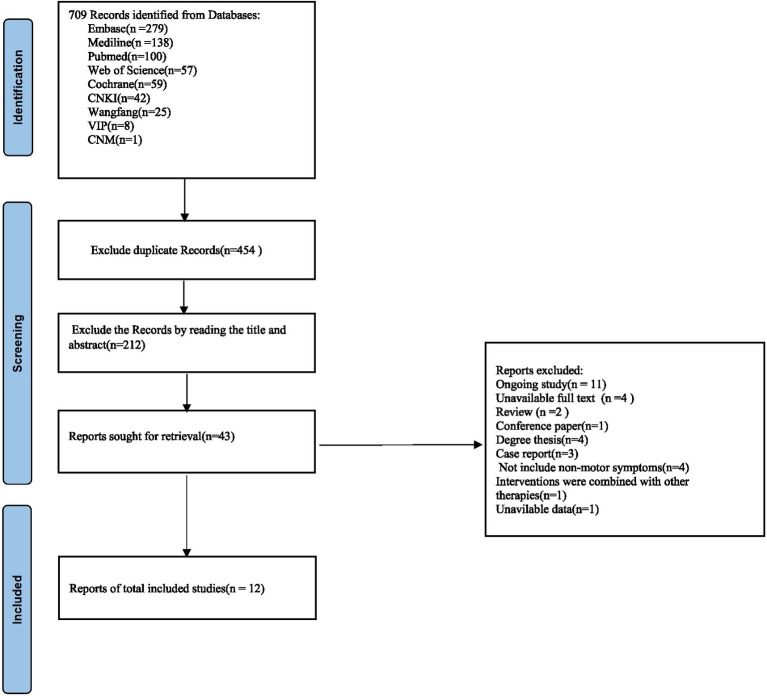
Flowchart of literature screening for meta-analysis. *Consider, if feasible to do so, reporting the number of records identified from each database or register searched (rather than the total number across all databases/registers). **If automation tools were used, indicate how many records were excluded by a human and how many were excluded by automation tools. Source: Page et al. (56).

Figure 1 displays the PRISMA flowchart for literature retrieval and selection. A total of 709 studies were retrieved, of which 454 duplicates were deleted. After title and abstract screening, 212 studies were excluded. Finally 12 studies were included in the study.

### 3.2 Characteristics of the included studies

The authors, publication year, region, patient characteristics, and intervention measures of the experimental and control groups are provided in Tables 1, 2.

**Table 1:** Demographic and clinical data in study.

Author, year	Country	Study type	Sample size		SEX	Mean age		Disease duration		Form of intervention	Intervention group (timing, duration, frequency, cycle, light, intensity)	CG (timing, Duration, Frequency, Cycle, light, intensity)	Distance		Instruments
			BLT	CG	M/F	BLT	CG	BLT	CG				BLT	CG	
Endo et al. (2020) (24)	Japan	NRCT	16	NA	6/10	66.2	NA	11	NA	Table-top illuminator	Evening,1 h, once a day, 12 weeks, bright white light, 5000lux	NA	50 cm		ESS/PDSS-2
Yun et al. (2022) (25)	China	NRCT	23	NA	14/9	64.9	NA	5.25	NA	Light box	Morning, 1 h, once a day, 1 week, bright white light, 10000lux	NA	≤86 cm		ESS/PSQI/PDSS-2/MOCA/PDSS-2/HAMD
Yoo et al. (2024) (26)	USA	NRCT	20	NA	8/12	72.1	NA	9	NA	Floor lamps or portable light therapy glasses	Morning, 2 h, once a day, 4 weeks, warm white light 400lux or green light CS>0.3	NA	46 cm		ESS/PDSS-2/FACIT-F/HDRS/HAMA/AS
Paus et al. (2007) (27)	Germany	RCT	18	18	23/13	63.6	63.4	7.4	7.9	Light boxes	Morning, 30 min, once a day, 15 day, bright white light, 7500lux	morning, 30 min, once a day, 15 day, placebo, 900lux	20 cm	100 cm	UPDRS-II/BDI/ESS
Sonja et al. (2019) (30)	Holland	RCT	35	37	32/40	58.9	65.8	NA	NA	Light box	Morning, 30 min, twice a day, 12 weeks, daylight spectrum light, 10000lux	morning, 30 min, twice a day, 12w, dim light, 200lux	30 cm	40 cm	HDRS/MMSE/SCOPA-SLEEP/WHO-QOL/GDS-30
Willis et al. (2018) (35)	Australia	RCT	10	10	12/8	70.8	66.9	NA	NA	Fluorescent tube	Evening, 1 h, once a day, 2 weeks, Polychromatic Light, 3000lux	evening, 1 h, once a day, 2w, red light, 200 lux	80-100 cm	80-100 cm	PDQ-39-ADL/PDQ-39 Social
Videnovic et al. (2017) (28)	USA	RCT	16	15	13/18	62.31	64.07	5.94	8.38	Light box	Morning & afternoon, 1 h, twice a day, 2 weeks, bright white light, 10,000 lux	morning& afternoon, 1 h, twice a day, 2w, dim-red light, < 300 lux	86.4 cm	86.4 cm	BDI/ESS/PDQ-39/PDSS/PSQI
Xie et al. (2025) (34)	China	RCT	22	20	14/8	63.82	NA	5.14	NA	Light box	Morning &afternoon, 1 h, twice a day, 30 day, bright white light, 10,000 lux	morning& afternoon, 1 h, twice a day, 30 day, dim light, 200 lux	63 cm	63 cm	ESS/FSS/HAMA-14/HAMD-24/MOCA/NMSQ/PDQ-39/PDSS-2/PSQI/RBDQ-HK/SCOPA-AUT/MDS-UPDRSII
Xiaoping (2025) (33)	China	RCT	H29	28	H 28/29	H 68.41	67.36	H 5.379	5.488	Light box	Afternoon, 30 min, once a day, 30 day, White light, 10000lux	Conventional treatment and care	50 cm	50 cm	PDSS
			M29		M 26/31	M 69.52		M 5.445			Afternoon, 30 min, once a day, 30 day, White light, 5000lux				
			L30		L 31/27	L 67.53		L5.505			Afternoon, 30 min, once a day, 30 day, White light, 1000ux				
Raymackers et al. (2019) (29)	Belgium	RCT	8	8	6/10	66.5	68.88	2.77	2.77	Head-mounted device	Morning, 45 min, once a day, 30 day, blue light, 472.7lux	morning, 45 min, once a day, 30 day, orange light, 175lux	NA	NA	HADS/ESS/FIS
Wyman et al. (2025) (32)	USA	RCT	44	47	62/29	70.2	65.9	NA	NA	Table-top device	Evening, 1 h, once a day, 6 month, blue and green light, 950lux	evening, 1 h, once a day, 6 month, White light, 100lux	80 cm	80 cm	ESS/BDI-II/PDQ-39/PDSS-2/BAI/MDS-UPDRSII
Videnovic et al. (2025) (31)	USA	RCT	OD 37	37	77/36	67	68	6.31	6.5	Light box	Evening, 1 h, once a day, 8 weeks, white light, 10000lux	morning& evening, 1 h, twice a day, 8w, 300lux	NA	NA	PDSS-2/PFS-16
			TD 39			66					Morning & evening, 1 h, twice a day, 8w, white light, 10000lux				

RCT: Randomized Controlled Trial; NRCT: Non-randomized controlled trial; NA: Not reported; BLT: bright light therapy; CG: Control group; BDI: Beck Depression Inventory; HDRS: Hamilton Depression Rating Scale; GDS-30:30-item Geriatric Depression Rating Scale; HAMD: Hamilton Depression Rating Scale; HAMD-24:24-item Hamilton Depression Scale; BDI-II: Beck Depression Inventory II; HAMA: Hamilton Anxiety Rating Scale; HAMA-14:14-item Hamilton Anxiety Scale; HADS-anxiety: The Hospital Anxiety and Depression Scale; MMSE: Mini-Mental State Examination; MoCA: Montreal Cognitive Assessment; FACIT-F: Functional Assessment of Chronic Illness Therapy – Fatigue Scale; FIS: Fatigue Impact Scale; FSS: Fatigue Severity Scale; ESS: Epworth Sleepiness Scale; PDSS: Parkinson’s Disease Sleep Scale; PSQI: Pittsburgh Sleep Quality Index; RBDQ-HK: RBD questionnaire-Hong Kong; SCOPA-SLEEP: Scales for Outcomes in Parkinson’s Disease–Sleep; PDQ-39: Parkinson’s Disease Questionnaire (39-item); WHO-QOL: World Health Organization Quality of Life assessment; UPDRSII: Unified Parkinson’s Disease Rating Scale; MDS-UPDRSII: Movement Disorder Society Sponsored Revision of the Unified Parkinson’s Disease Rating Scale. PFS-16: Parkinson’s Disease Fatigue Scale; BAI: Beck anxiety inventory; H: high intensity; M: Medium intensity; L: Low intensity; OD: once daily BLT group; TD: twice daily BLT group.

**Table 2:** Description of the assessment tools included in the study.

Outcome indicators		Related study	Relevant scales
Depression	8RCTs 2NRCTs	Paus et al. (2007) (27) and Videnovic et al. (2017) (28)	Beck Depression Inventory (BDI)
		Yoo et al. (2024) (26) and Sonja et al. (2019) (30)	Hamilton Depression Rating Scale (HDRS)
		Yun et al. (2022) (25)	Hamilton Depression Rating Scale (HAMD)
		Xie et al. (2025) (34)	24-item Hamilton Depression Scale (HAMD-24)
		Willis et al. (2018) (35) and Wyman et al. (2025) (32)	Beck Depression Inventory II (BDI-II)
		Sonja et al. (2019) (30)	30-item Geriatric Depression Rating Scale (GDS-30)
		Raymackers et al. (2019) (29)	Hospital Anxiety and Depression Scale (HADS)
Anxiety	1NRCT 3RCTs	Xie et al. (2025) (34)	14-item Hamilton Anxiety Scale (HAMA-14)
		Raymackers et al. (2019) (29)	Hospital Anxiety and Depression Scale (HADS)
		Wyman et al. (2025) (32)	Beck anxiety inventory (BAI)
		Yoo et al. (2024) (26)	Hamilton Anxiety Rating Scale (HAMA)
Cognition	1NRCT 2RCTs	Xie et al. (2025) (34) and Yun et al. (2022) (25)	Montreal Cognitive Assessment (MoCA)
		Sonja et al. (2019) (30)	Mini-Mental State Examination (MMSE)
Fatigue	1NRCT 4RCTs	Raymackers et al. (2019) (29)	Fatigue Impact Scale (FIS)
		Xie et al. (2025) (34) and Videnovic et al. (2017) (28)	Fatigue Severity Scale (FSS)
		Yoo et al. (2024) (26)	Functional Assessment of Chronic Illness Therapy – Fatigue Scale (FACIT-F)
		Videnovic et al. (2025) (31)	Parkinson’s Disease Fatigue Scale (PFS-16)
Daytime sleepiness	3NRCTs 6RCTs	Xie et al. (2025) (34), Videnovic et al. (2017) (28), Paus et al. (2007) (27), Raymackers et al. (2019) (29), Yoo et al. (2024) (26), Endo et al. (2020) (24), Yun et al. (2022) (25), and Wyman et al. (2025) (32)	Epworth sleepiness scale (ESS)
		Sonja et al. (2019) (30)	Scales for Outcomes in Parkinson’s Disease–Sleep (SCOPA-SLEEP)
Nighttime sleepiness	3NRCTs 6RCTs	Videnovic et al. (2025) (31), Yoo et al. (2024) (26), Endo et al. (2020) (24), Yun et al. (2022) (25), Wyman et al. (2025) (32), and Xie et al. (2025) (34),	Parkinson’s disease sleep scale 2 (PDSS-2)
		Sonja et al. (2019) (30)	Scales for Outcomes in Parkinson’s Disease–Sleep (SCOPA-SLEEP)
		Xie et al. (2025) (34)	RBD questionnaire-Hong Kong (RBDQ-HK)
		Xiaoping (2025) (33) and Videnovic et al. (2017) (28),	Parkinson’s Disease Sleep Scale (PDSS)
Sleep quality	1NRCT 3RCTs	Sonja et al. (2019) (30)	Scales for Outcomes in Parkinson’s Disease–Sleep (SCOPA-SLEEP)
		Videnovic et al. (2017) (28), Xie et al. (2025) (34), Sonja et al. (2019) (30), and Yun et al. (2022) (25)	Pittsburgh Sleep Quality Index (PSQI)
Quality of life	8RCTs	Willis et al. (2018) (35), Wyman et al. (2025) (32), Videnovic et al. (2017) (28), and Xie et al. (2025) (34),	Parkinson’s Disease Questionnaire (39-item) (PDQ-39)
		Sonja et al. (2019) (30)	World Health Organization Quality of Life assessment (WHO-QOL)
		Paus et al. (2007) (27) and Wyman et al. (2025) (32)	Unified Parkinson’s Disease Rating Scale (UPDRSII)
		Xie et al. (2025) (34)	Movement Disorder Society Sponsored Revision of the Unified Parkinson’s Disease Rating Scale (MDS-UPDRSII)

#### 3.2.1 Participants

59 patients from three NRCT trials (24–26) and 537 participants from nine RCT studies (27–35) comprised the total of 596 PD patients, whose ages ranged from 58.9 to 72.1 years. The mean disease duration varied from 2.77 to 11 years (24, 29). All enrolled patients were in a stable phase of pharmacological treatment.

#### 3.2.2 Intervention measures

BLT was used as the experimental group’s intervention in all investigations, and it was given to patients in their homes or in hospitals. Seven studies used light boxes (25, 27, 28, 30, 31, 33, 34), two used table devices (24, 32), one combined lamps with portable light therapy glasses (26), one used fluorescent tubes (35), and one used head-mounted devices (29). Interventions were conducted in the morning, afternoon, or evening, and lasted between 30 min to 2 h, and frequency ranging from once to twice daily. One study compared weekly BLT sessions to examine frequency-dependent effects (31). The intervention cycle spanned 1 to 24 weeks, with light intensity ranging from 400 lux to 10,000 lux; six studies specifically employed 10,000 lux (26, 28, 30, 31, 33, 34).

#### 3.2.3 Control measures

Of the nine included RCTs, eight used placebo interventions for the control group (27–30, 34, 35), mainly dim light therapy. In one of these eight studies, the control group was also given BLT (the same intensity as the experimental group and low-frequency) (31). To determine the optimal light intensity, this study excluded the treatment data of the control group that used the same light intensity but different light frequencies. A meta-analysis was conducted on the data of high-intensity and low-intensity light treatments (31). One RCT used conventional medication or care in the control group (33).

#### 3.2.4 Quality evaluation of the included literature

**Figure 2:**
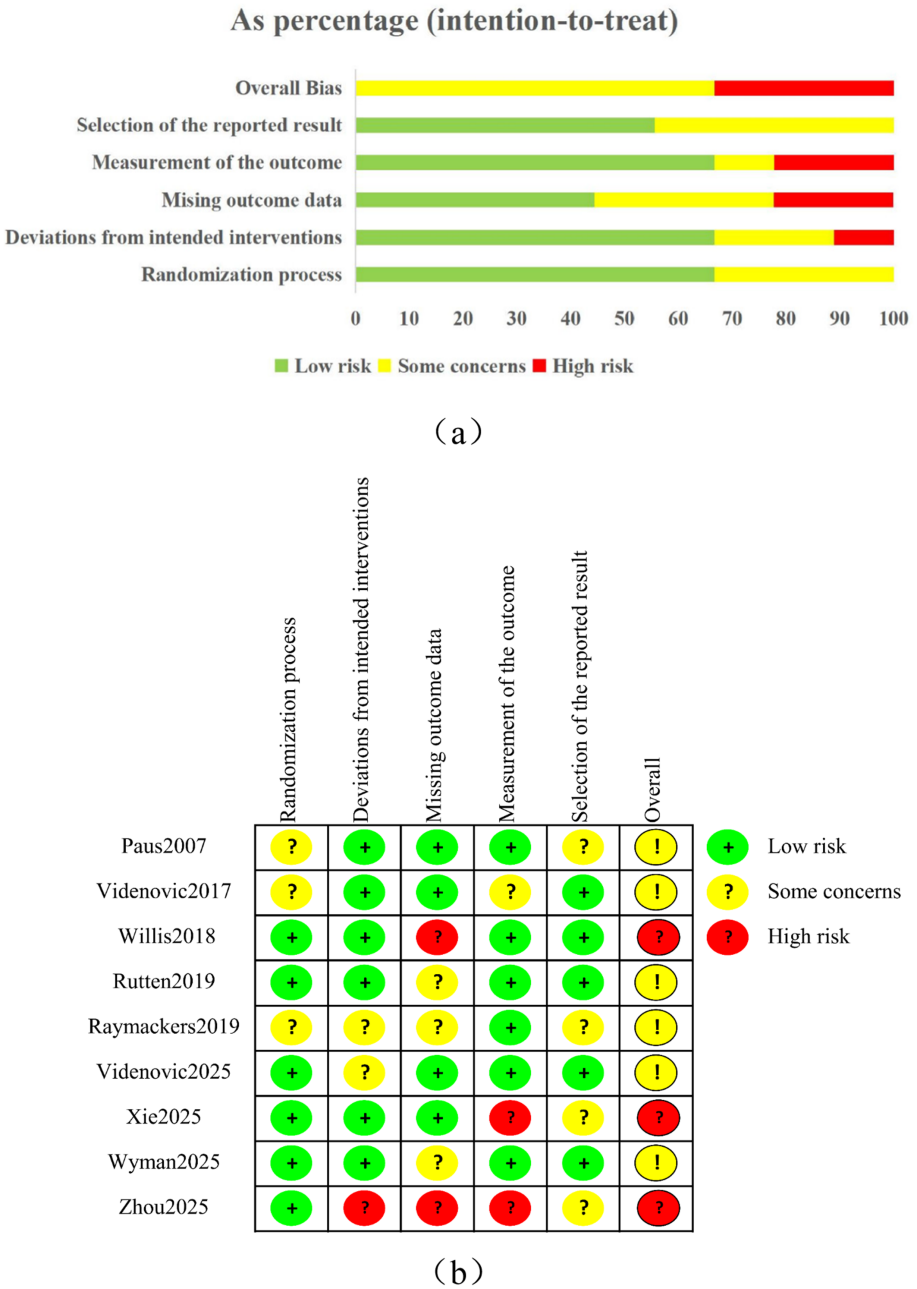
Risk of bias assessment was conducted using ROB2: **(a)** overall risk of bias; **(b)** risk of bias in a single trial.

The ROB2 tool was used to evaluate the quality of the nine RCTs. Figure 2 illustrates that two studies had high risk in missing outcome data, two in outcome measurement, four in selective reporting, three in random sequence creation, and three in deviation from intended interventions. Overall, there was a moderate risk of bias. For the three NRCTs evaluated with ROBINS-I V2, all showed an overall high risk of bias, as shown in Table 3.

**Table 3:** Bias risk assessment was conducted using ROBINS-I V2.

Study	Bias due to confounding	Bias in selection of participants into the study	Bias in classification of interventions	Bias due to deviations from intended interventions	Bias due to missing data	Bias in measurement of outcomes	Bias in selection of the reported result	Overall risk of bias
Endo et al. (2020) (24)	Serious	Low	Low	Low	Low	Low	Low	Serious
Yun et al. (2022) (25)	Serious	Low	Low	Serious	Serious	Moderate	Low	Serious
Yoo et al. (2024) (26)	Serious	Low	Low	Moderate	Serious	Moderate	Low	Serious

### 3.3 Effect of BLT on non-motor symptoms in PD patients

Only change data of patients’ symptom scores before and after treatment could be collected from some studies due to differences in data types between research (31, 32). NRCTs had no control group data since they lacked a control therapy. Three comparison methods are applicable to various types of research designs, effectively addressing the problem of a lack of control groups in studies like NRCTs and adapting to the reality of inconsistent data structures. In this meta-analysis, data analyses were conducted on mean change in pre- and post- control group vs. mean change in pre- and post- BLT, pre- and post- BLT, pre- and post- control group. Three comparison methods are applicable to different types of research designs, effectively addressing the issue of a lack of control groups in studies such as NRCTs, and adapting to the reality of inconsistent data structures. This method system can more comprehensively evaluate the effectiveness of BLT: it can not only determine whether BLT treatment improves patients’ non-motor symptoms, but it can also ascertain whether it is more effective than traditional treatment and care measures and whether it is noticeably better than the control group’s dim light treatment.

#### 3.3.1 Effect of BLT on depression in PD patients

**Figure 3:**
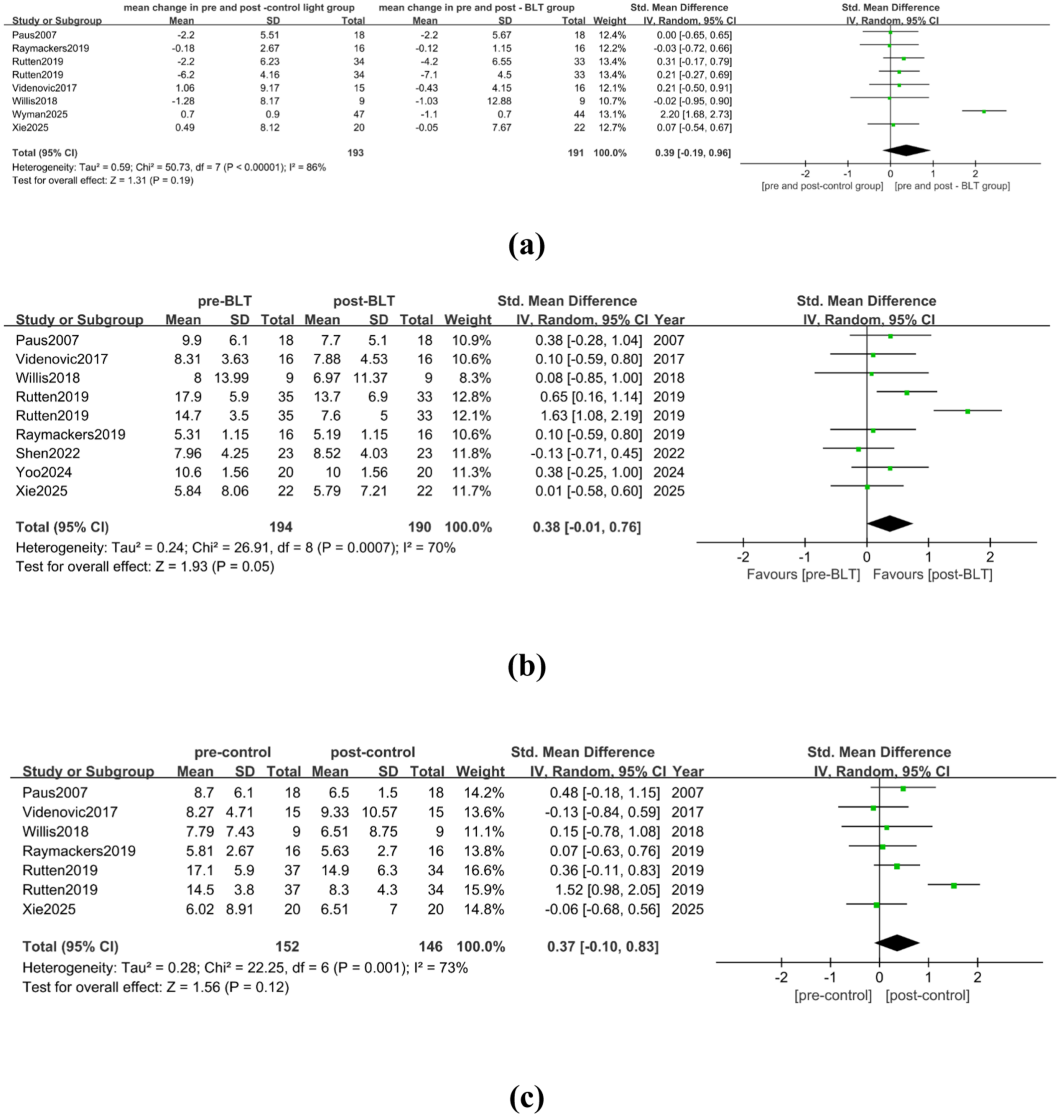
The therapeutic effect of BLT on depression in PD patients. **(a)** mean change in pre-and post-control group vs. mean change in pre- and post-BLT, **(b)** pre- and post-BLT, **(c)** pre-and post-control group.

Seven trials with a total of 193 patient data were included in the meta-analysis of BLT for depression symptoms in patients with PD. There was significant heterogeneity among the studies (I^2^ = 86%). No statistically significant difference was found in the change of depression scores between the control group and the BLT group (SMD = 0.39, 95% CI = [−0.19, 0.96]). The detailed results are shown in Figure 3a. After trimming and deleting one article (32), the heterogeneity among the studies decreased (I^2^ = 0%), but the research results remained unchanged (SMD = 0.14, 95% CI = [−0.09, 0.37]), as shown in Supplementary Figure 1.

Eight studies with 194 PD patients were included in the meta-analysis after the NRCT data was incorporated. The results showed high heterogeneity among the studies (I^2^ = 70%), and BLT did not show a statistically significant effect on the depression of PD patients (SMD = 0.38, 95% CI = [−0.01, 0.76]), as shown in Figure 3b. Following the removal of one article (30), Supplementary Figure 2 indicates that BLT helped alleviate PD patients’ depression (SMD = 0.23, 95% CI = [0.01, 0.45]), and that there was no substantial heterogeneity among the trials (I^2^ = 0%).

When analyzing the effect of control intervention measures on the improvement of depression in PD patients, the meta-analysis included 6 studies involving 152 PD patients, and there was high heterogeneity among the studies (I^2^ = 73%). The control intervention did not significantly alleviate depression of PD patients (SMD = 0.37, 95% CI [−0.10, 0.83]), as shown in Figure 3c. After trimming and deleting one article (30), there was no significant heterogeneity among the studies (I^2^ = 0%), and the results did not change (SMD = 0.18, 95% CI = [−0.08, 0.44]), as shown in Supplementary Figure 3.

#### 3.3.2 Effect of BLT on anxiety in PD patients

**Figure 4:**
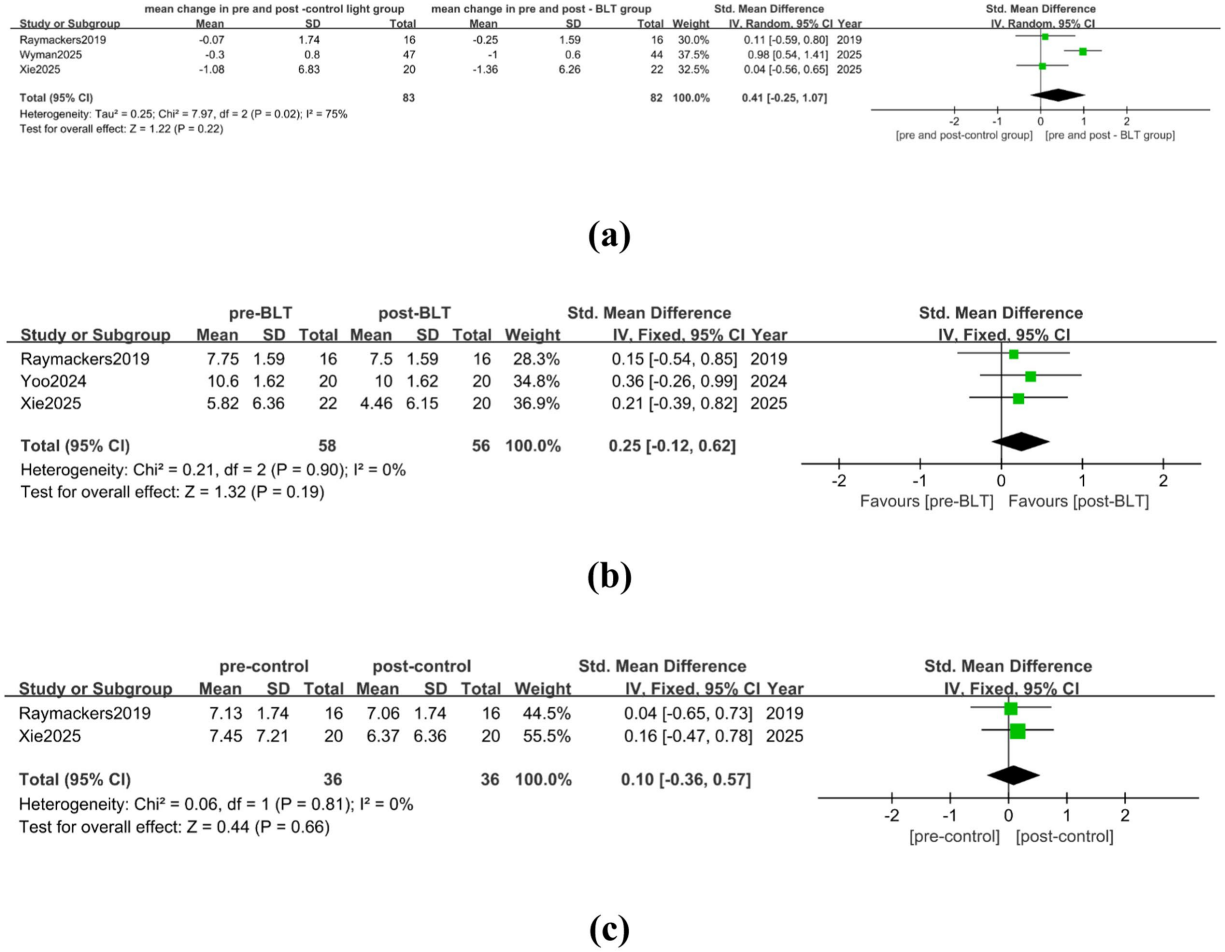
The therapeutic effect of BLT on anxiety in PD patient. **(a)** mean change in pre-and post-control group vs. mean change in pre- and post-BLT, **(b)** pre- and post-BLT, **(c)** pre-and post-control group.

Three trials with 83 valid data points were included in the meta-analysis, and the heterogeneity was high (I^2^ = 75%). Figure 4a illustrates that BLT did not significantly reduce PD patients’ anxiety as compared to the control group (SMD = 0.41, 95% CI = [−0.25, 1.07]). There was no significant heterogeneity across the trials following the trimming and deletion of one article (32), there was no significant heterogeneity among the studies (I^2^ = 0%). The finding that BLT was not better than the control group in reducing PD patients’ anxiety remained (SMD = 0.07, 95% CI = [−0.39, 0.53]), as shown in Supplementary Figure 4. Three trials totaling 58 PD patients were included in the meta-analysis. Compared with the baseline, BLT showed no significant statistical difference in alleviating anxiety in patients with PD (SMD = 0.25, 95% CI = [−0.12, 0.62]), as shown in Figure 4b. The treatment of the control group could not alleviate the anxiety symptoms of PD patients (SMD = 0.10, 95% CI = [−0.36, 0.57]). The specific results are shown in Figure 4c.

#### 3.3.3 Effect of BLT on the cognition of PD patients

**Figure 5:**
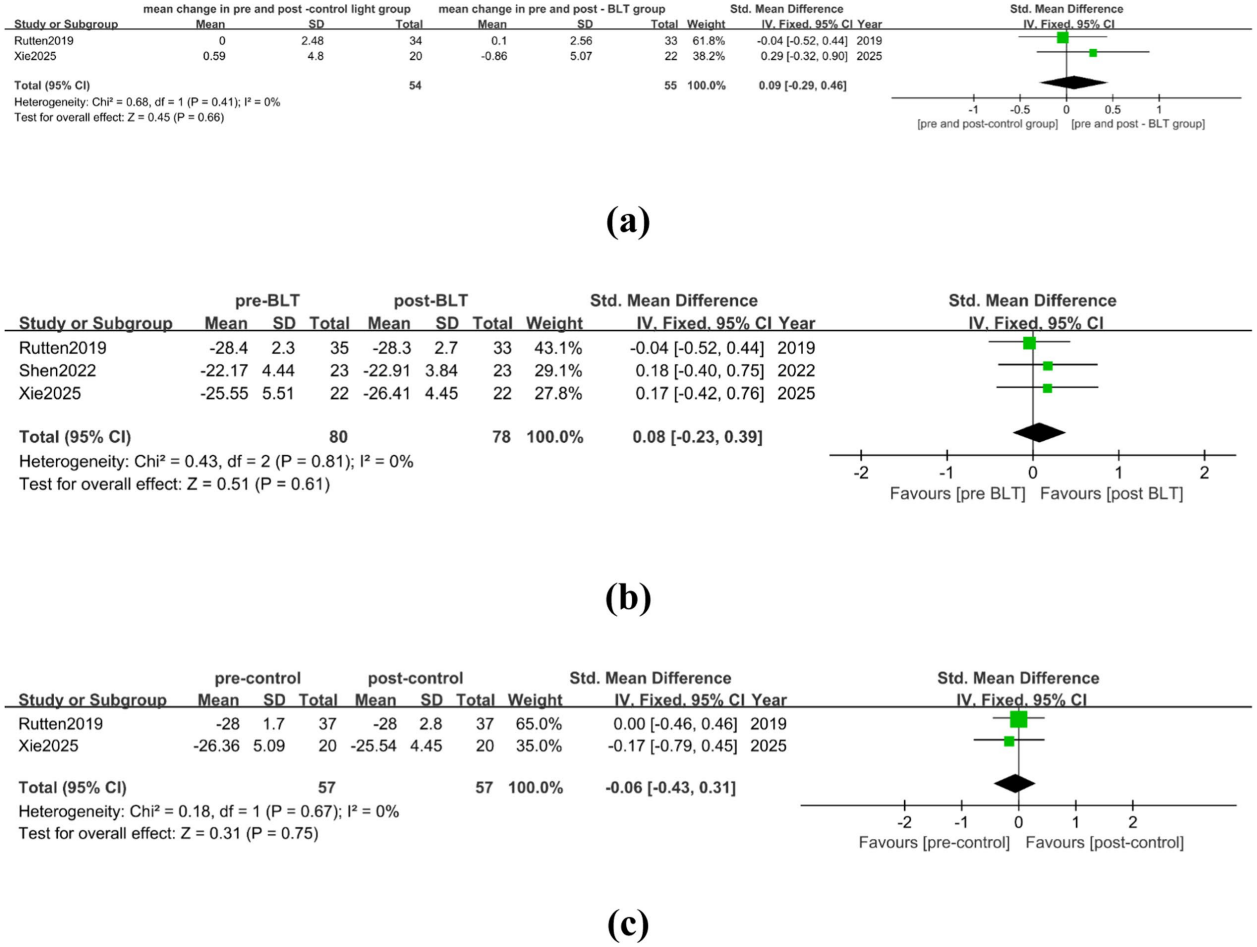
The therapeutic effect of BLT on cognition of PD patients. **(a)** mean change in pre-and post-control group vs. mean change in pre- and post-BLT, **(b)** pre- and post-BLT, **(c)** pre-and post-control group.

In the meta-analysis, two RCTs were included, involving 54 valid data, and there was low heterogeneity among the studies (I^2^ = 0%). BLT did not significantly improve the cognitive function of PD patients as compared to the control group treatment (SMD = 0.09, 95% CI = [−0.29, 0.46]), as shown in Figure 5a. 80 patients with PD were included in the three trials that made up the meta-analysis of BLT’s impact on patients’ improved cognitive performance. As seen in Figure 5b, BLT did not significantly improve the patients’ cognition (SMD = 0.08, 95% CI = [−0.23, 0.39]). As seen in Figure 5c, the dim light treatment in the control group was unable to improve the patients’ cognitive function and had a tendency to worsen the cognitive impairment of PD patients (SMD = -0.06, 95% CI = [−0.43, 0.31]).

#### 3.3.4 Effect of BLT on the fatigue of PD patients

**Figure 6:**
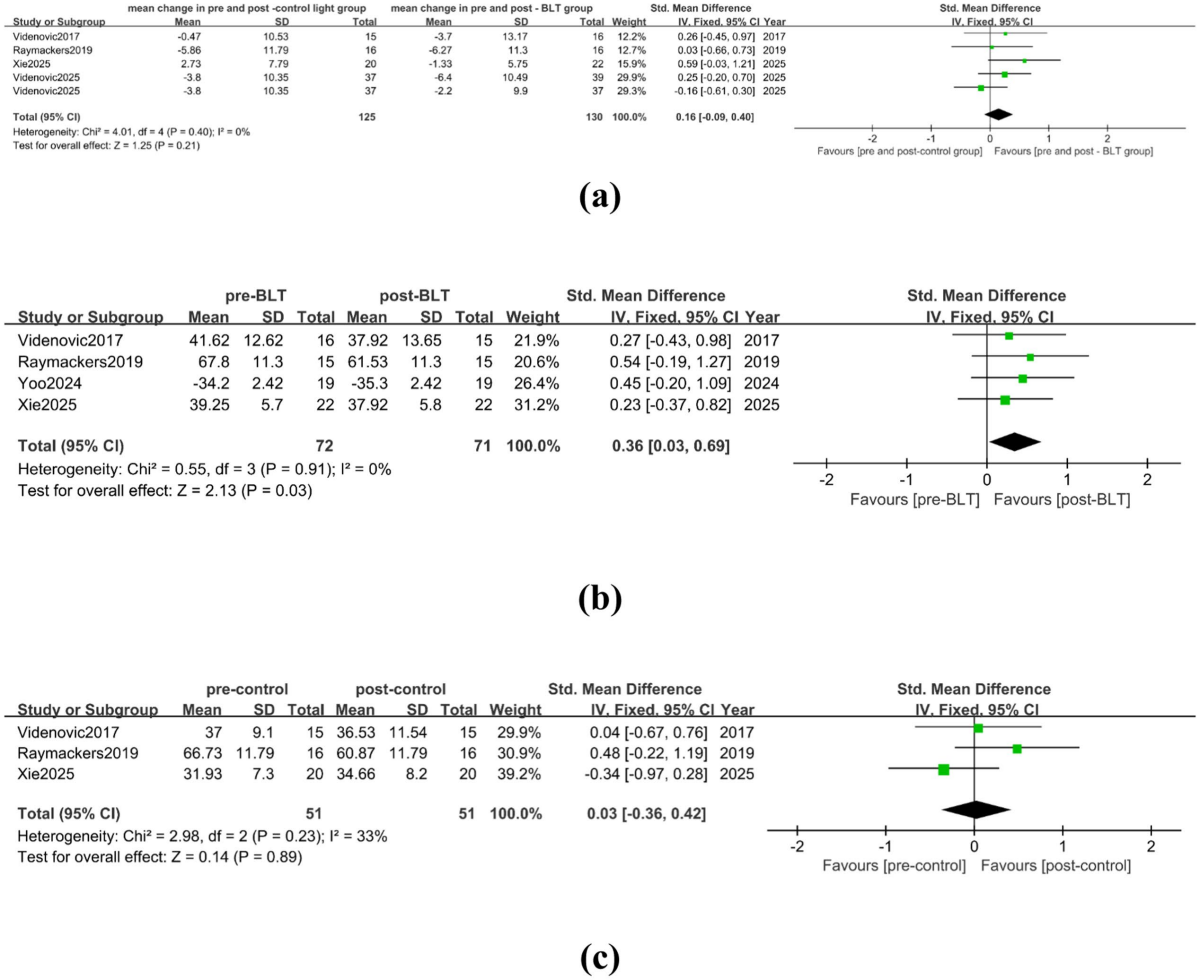
Effect of BLT on fatigue in PD patients. **(a)** mean change in pre-and post-control group vs. mean change in pre- and post-BLT, **(b)** pre- and post-BLT, **(c)** pre-and post-control group.

In the meta-analysis comparing BLT with the control group, there were 4 studies included, involving data from 125 patients. The studies’ heterogeneity was low (I^2^ = 0%), and Figure 6a indicates that BLT did not significantly reduce PD patients’ fatigue as compared to the control group (SMD = 0.16, 95% CI [−0.09, 0.40]). A total of 72 patients were included in the four studies, according to the analysis results pre- and post- BLT treatment, the heterogeneity among the studies was low (I^2^ = 0%), BLT significantly reduced the patients’ fatigue symptoms when compared to the baseline (SMD = 0.36, 95% CI = [0.03, 0.69]), as shown in Figure 6b. No alleviation in fatigue symptoms of PD patients was found following the control group therapy (SMD = 0.03, 95% CI = [−0.36, 0.42]), as shown in Figure 6c.

#### 3.3.5 Effect of BLT on daytime sleepiness in PD patients

**Figure 7:**
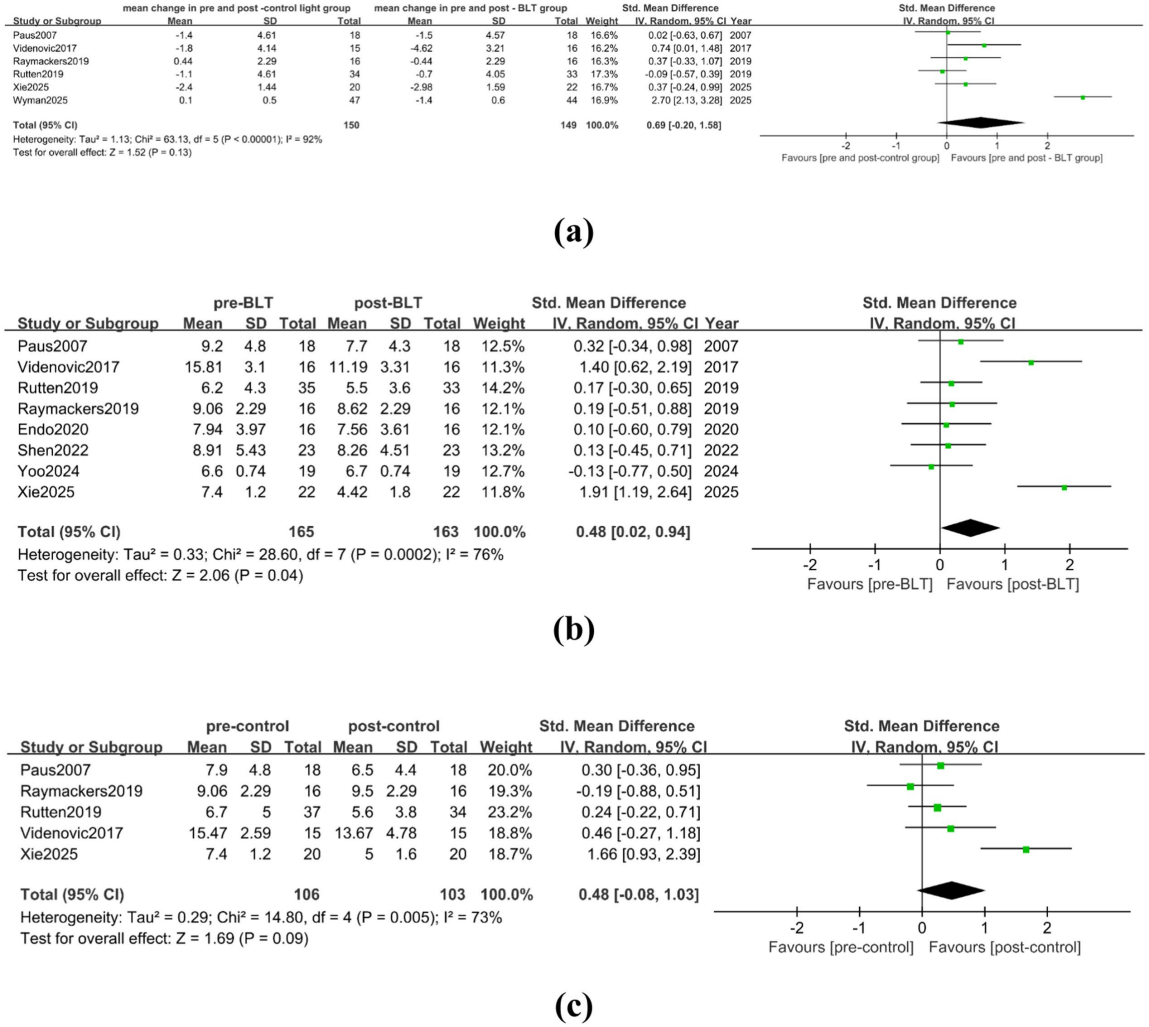
Effect of BLT on daytime sleepiness in PD patients. **(a)** mean change in pre-and post-control group vs. mean change in pre- and post-BLT, **(b)** pre- and post-BLT, **(c)** pre-and post-control group.

In the meta-analysis comparing BLT with the control group, a total of 6 studies were included, with 150 valid data points. There was high heterogeneity among the studies (I^2^ = 92%). BLT did not significantly reduce PD patients’ daytime sleepiness when compared to the control group (SMD = 0.69, 95% CI = [−0.20, 1.58]), as shown in Figure 7a. After trimming and deleting one study (I^2^ = 9%) (32), BLT did not significantly reduce patients’ daytime sleepiness when compared to the control group treatment (SMD = 0.21, 95% CI = [−0.06, 0.49]), as shown in Supplementary Figure 5. The meta-analysis results pre- and post- BLT intervention showed high heterogeneity among the studies (I^2^ = 76%), and BLT did significantly reduce daytime sleepiness of PD patients (SMD = 0.48, 95% CI = [0.02, 0.94]), as shown in Figure 7b. After trimming and deleting one article (34), the heterogeneity decreased (I^2^ = 41%), and the results showed that BLT had a significant statistical significance in reducing daytime sleepiness of PD patients (SMD = 0.25, 95% CI = [0.01, 0.48]), as shown in Supplementary Figure 6. There was no statistically significant relief in daytime sleepiness compared to the baseline in patients following the control intervention (SMD = 0.48, 95% CI = [−0.08, 1.03]) (see Figure 7c).

#### 3.3.6 Effect of BLT on nighttime sleep in PD patients

**Figure 8:**
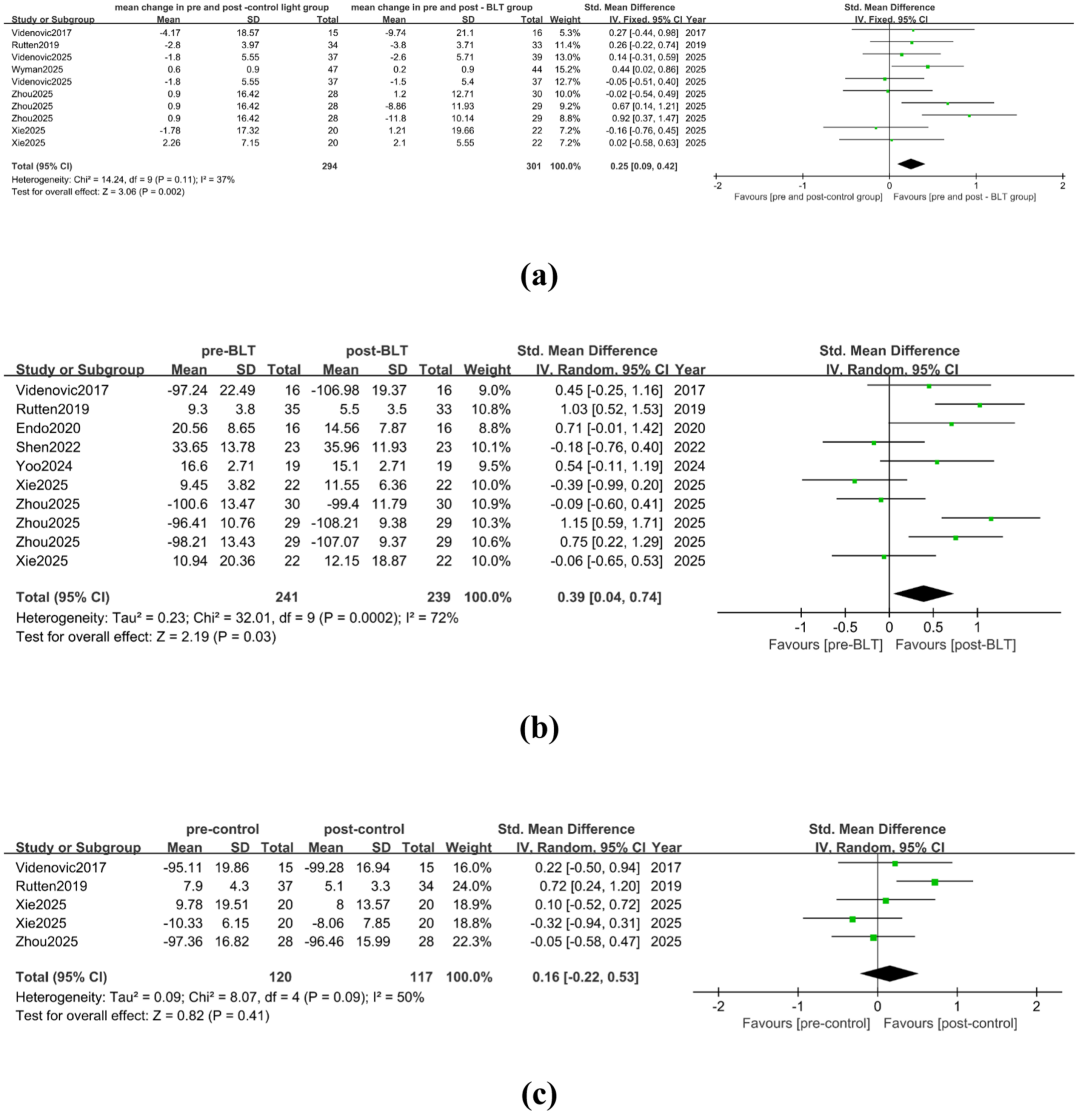
Effect of BLT on nighttime sleep in PD patients. **(a)** mean change in pre-and post-control group vs. mean change in pre- and post-BLT, **(b)** pre- and post-BLT, **(c)** pre-and post-control group.

In the meta-analysis comparing BLT with the control treatment, there were 6 studies included, with a total of 294 valid data. The heterogeneity among the studies was low (I^2^ = 37%). Compared with the control group treatment, BLT showed an advantage in alleviating nighttime sleep of PD patients (SMD = 0.25, 95% CI = [0.09, 0.42]), as shown in Figure 8a. Eight trials with a total of 241 valid data were included in the meta-analysis conducted pre- and post- BLT treatment. The heterogeneity among the studies was high (I^2^ = 72%), and BLT significantly improved nighttime sleep in PD patients (SMD = 0.39, 95% CI = [0.04, 0.74]), as shown in Figure 8b. PD patients’ nighttime sleep was not improved by the control group’s treatment (SMD = 0.16, 95% CI = [−0.22, 0.53]), as shown in Figure 8c.

#### 3.3.7 Effect of BLT on sleep quality in PD patients

**Figure 9:**
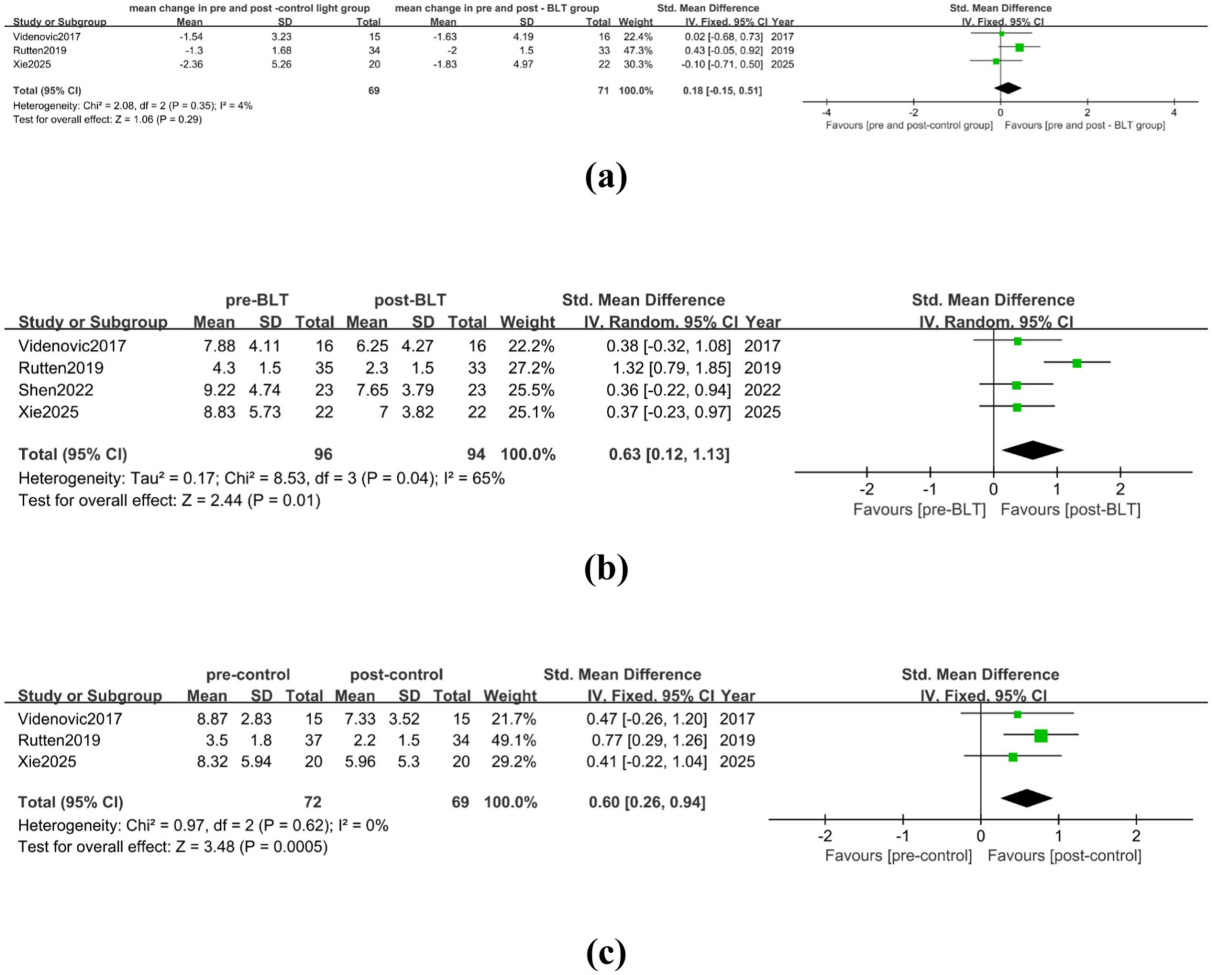
Effect of BLT on the sleep quality of PD patients. **(a)** mean change in pre-and post-control group vs. mean change in pre- and post-BLT, **(b)** pre- and post-BLT, **(c)** pre-and post-control group.

In the meta-analysis comparing BLT with the control group, a total of 3 studies were included, involving 69 patients with valid data. The heterogeneity among the studies was low (I^2^ = 4%). As seen in Figure 9a, BLT did not significantly improve the quality of sleep for Parkinson’s disease patients as compared to the control group (SMD = 0.18, 95% CI = [−0.15, 0.51]). The meta-analysis pre- and post-BLT showed that the heterogeneity among the studies was high (I^2^ = 65%), and BLT significantly improved the sleep quality of patients with Parkinson’s disease (SMD = 0.63, 95% CI = [0.12, 1.13]), as shown in Figure 9b. After deleting one study, there was no significant heterogeneity among the studies (I^2^ = 0%), and the results were in line with the previous findings (SMD = 0.37, 95% CI = [0.01, 0.73]) (Supplementary Figure 7). As seen in Figure 9c, the control group’s sleep quality also changed statistically significantly from the baseline (SMD = 0.60, 95% CI = [0.26, 0.94]).

#### 3.3.8 Effect of BLT on the quality of life of PD patients

**Figure 10:**
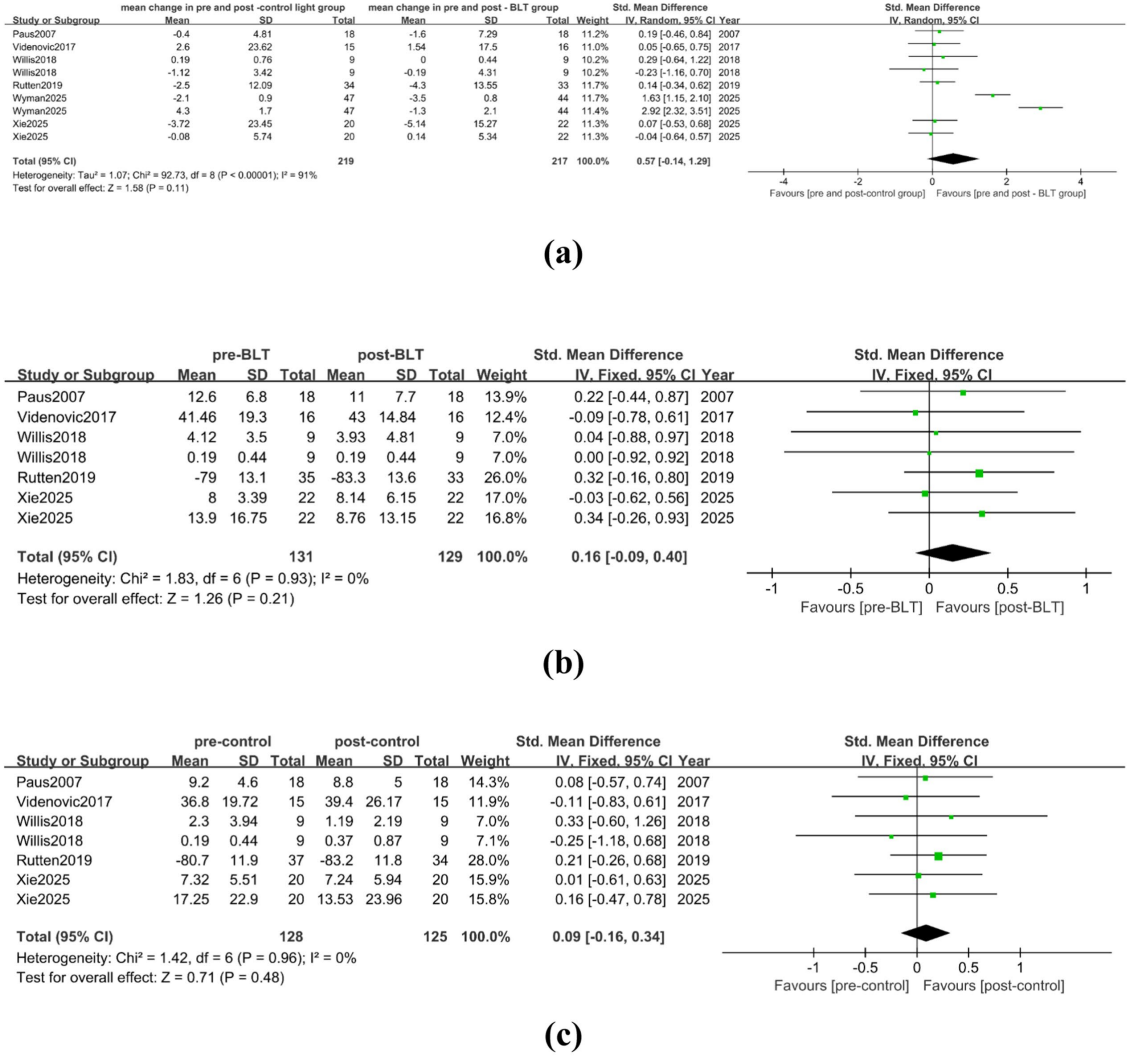
Effect of BLT on the quality of life of PD patients. (a) mean change in pre-and post-control group vs. mean change in pre- and post-BLT, **(b)** pre- and post-BLT, **(c)** pre-and post-control group.

In the meta-analysis comparing BLT with the control group, there were 6 studies included, involving 219 patients with valid data. There was a high degree of heterogeneity (I^2^ = 91%), and the results indicated that BLT did not significantly improve patients’ quality of life when compared to the control treatment (SMD = 0.57, 95% CI = [−0.14, 1.29]), as shown in Figure 10a. After trimming and deleting one article (32), there was no significant heterogeneity among the studies (I^2^ = 0%), but the results were consistent with the previous ones. According to Supplementary Figure 8, there was no statistically significant difference in the treatment effect of BLT and the control group in terms of improving patients’ quality of life (SMD = 0.08, 95% CI = [−0.17, 0.33]). In the meta-analysis pre- and post- BLT intervention, there were 5 studies involving 131 PD patients. There was no significant heterogeneity among the studies (I^2^ = 0%), and the results showed that BLT could not significantly improve the quality of life of PD patients (SMD = 0.16, 95% CI = [−0.09, 0.40]), as shown in Figure 10b. The control group also failed to improve the quality of life of Parkinson’s disease patients (SMD = 0.09, 95% CI = [−0.16, 0.34]), as shown in Figure 10c.

### 3.4 Subgroup analysis results

#### 3.4.1 Long-term effects of BLT on non-motor symptoms in PD

**Figure 11:**
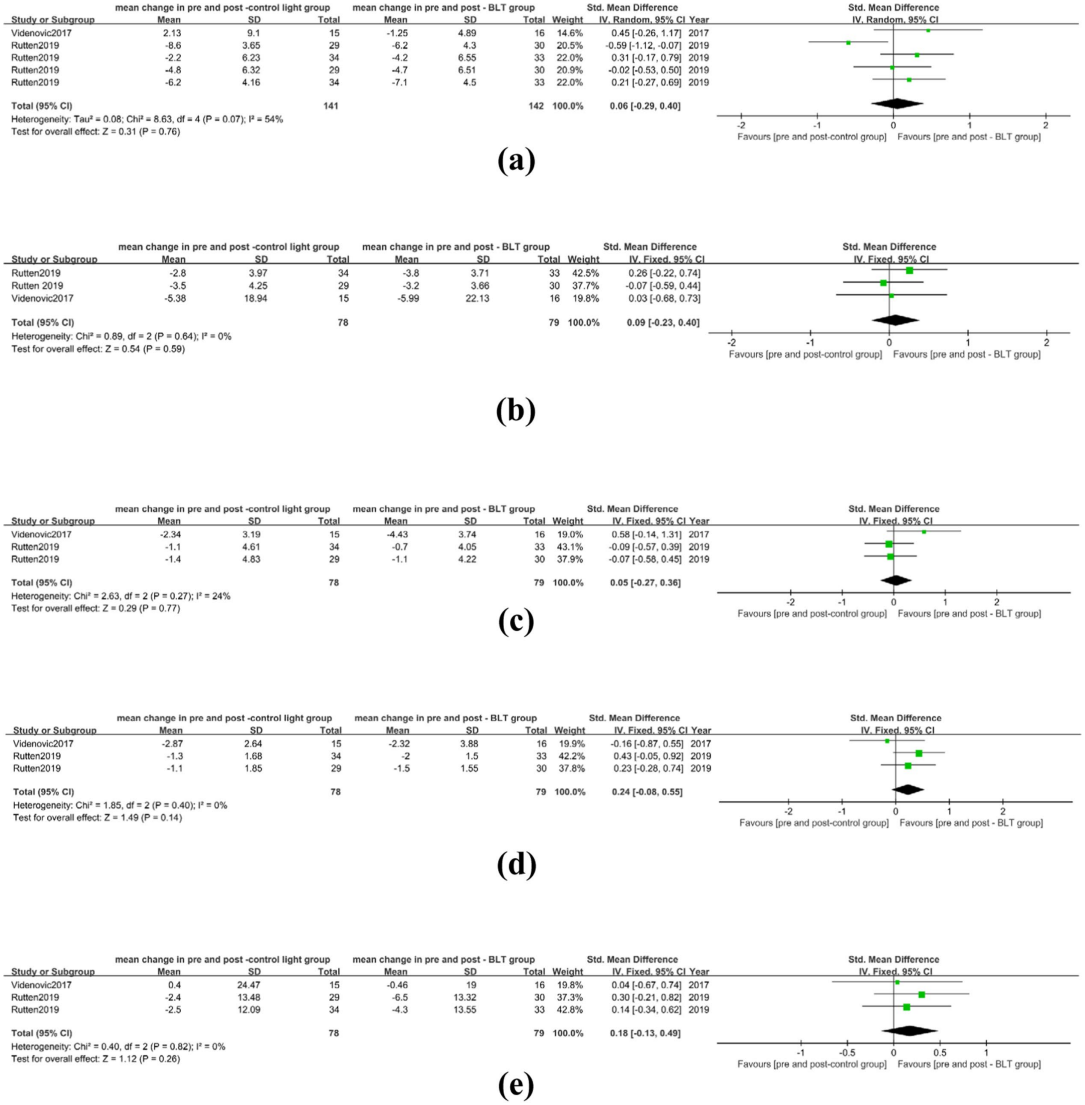
Long-term effects of BLT on non-motor symptoms in PD. **(a)** depression **(b)** nighttime sleep **(c)** daytime sleepiness **(d)** sleep quality **(e)** quality of life.

Two studies evaluated the improvement of symptoms one month or longer after treatment, with the longest follow-up period being six months (28, 30). From the perspective of long-term effects, compared with the control group’s treatment, BLT showed no significant advantage in improving depression of PD patients (SMD = 0.06, 95%CI = [−0.29, 0.40]), nighttime sleep (SMD = 0.09, 95% CI = [−0.23,0.40]), daytime sleepiness (SMD = 0.05, 95%CI = [−0.27, 0.36]), sleep quality (SMD = 0.24, 95%CI = [−0.08, 0.55]), and quality of life (SMD = 0.18, 95% CI = [−0.13, 0.49]), as shown in Figure 11. However, compared with the baseline levels of various non-motor symptoms of the patients, one month or longer after BLT treatment, there were still significant improvement effects on depression (SMD = 0.97, 95% CI = [0.48, 1.46]), nighttime sleep (SMD = 0.81, 95% CI = [0.49, 1.13]), sleep quality (SMD = 1.03, 95% CI = [0.70, 1.36]), and quality of life (SMD = 0.33, 95% CI = [0.02, 0.64]), Long-term follow-up revealed that BLT failed to alleviate daytime sleepiness in patients with PD (SMD = 0.46, 95% CI = [−0.06, 0.98]), as shown in Supplementary Figure 9.

#### 3.4.2 Cycle of BLT

**Figure 12:**
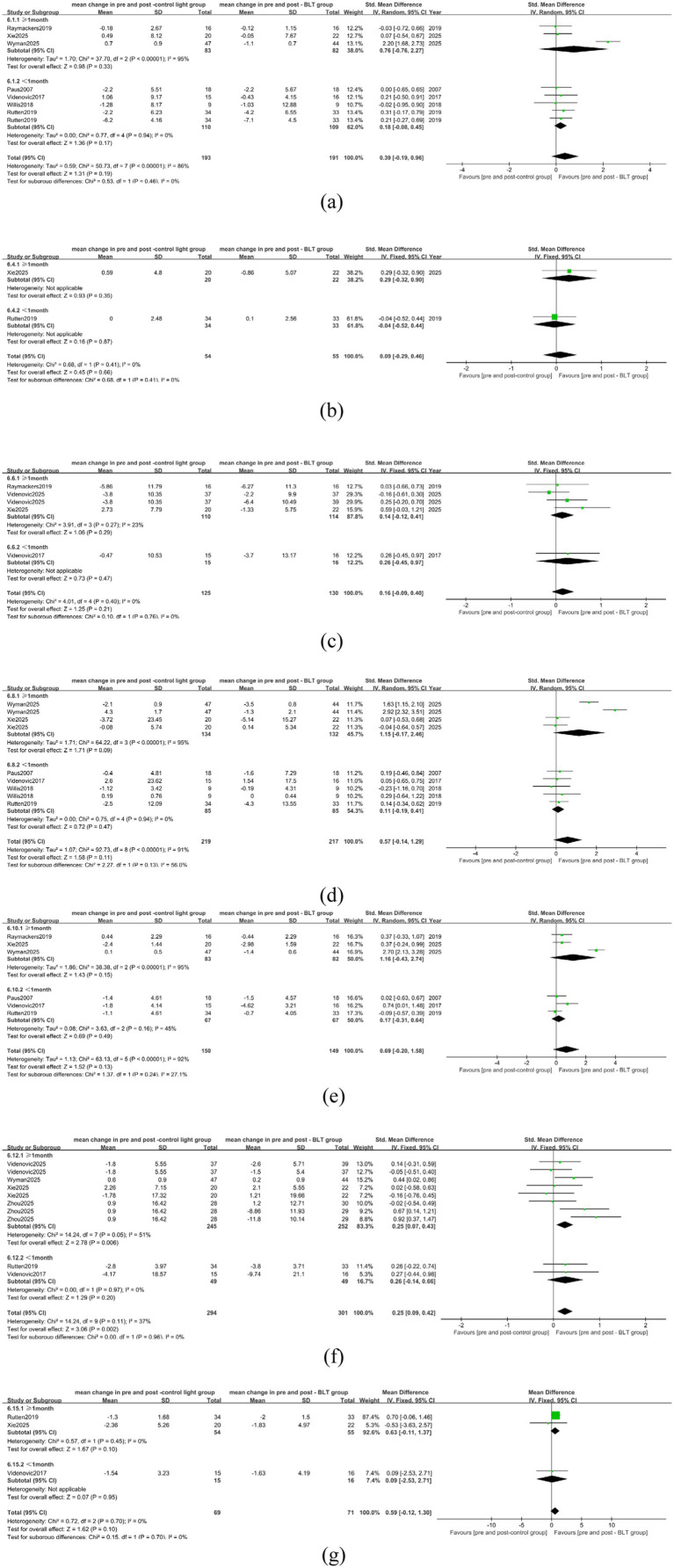
The BLT cycle affects the effect. **(a)** depression **(b)** cognition **(c)** fatigue **(d)** quality of life **(e)** daytime sleepiness **(f)** nighttime sleep **(g)** sleep quality.

Of the included studies, eight studies involved BLT treatment for at least one month (24, 26, 29–34). The comparison analysis of the results between the BLT group and the control group showed that the effects of the BLT group in alleviating depression (SMD = 0.39, 95% CI = [−0.19, 0.96]), cognitive function (SMD = 0.09, 95% CI = [−0.29, 0.46]), fatigue (SMD = 0.16, 95% CI = [−0.09, 0.40]), daytime sleepiness (SMD = 0.69, 95% CI = [−0.20, 1.58]), nighttime sleep (SMD = 0.25, 95% CI = [0.09, 0.42]), sleep quality (SMD = 0.59, 95% CI = [−0.12, 1.30]), and quality of life (SMD = 0.57, 95% CI = [−0.14, 1.29]) were not superior to those of the control group. Therefore, lengthening the intervention duration does not boost the therapeutic advantage of BLT, as shown in Figure 12.

However, when comparing the results after BLT treatment with the baseline condition, the study showed that it alleviated depression (SMD = 0.38, 95% CI = [−0.01, 0.76]), anxiety (SMD = 0.25, 95% CI = [−0.12, 0.62]), cognitive function (SMD = 0.08, 95% CI = [−0.23, 0.39]), fatigue (SMD = 0.36, 95% CI = [0.03, 0.69]), daytime sleepiness (SMD = 0.48, 95% CI = [0.02, 0.94]), nighttime sleep (SMD = 0.39, 95% CI = [0.04, 0.74]), sleep quality (SMD = 0.63, 95% CI = [0.12, 1.13]), and quality of life (SMD = 0.16, 95% CI = [−0.09, 0.40]) were not superior to those of the control group. As seen in Supplementary Figure 10, the therapeutic impact of BLT is therefore unaffected by whether the intervention period is longer than one month.

#### 3.4.3 Light source distance

**Figure 13:**
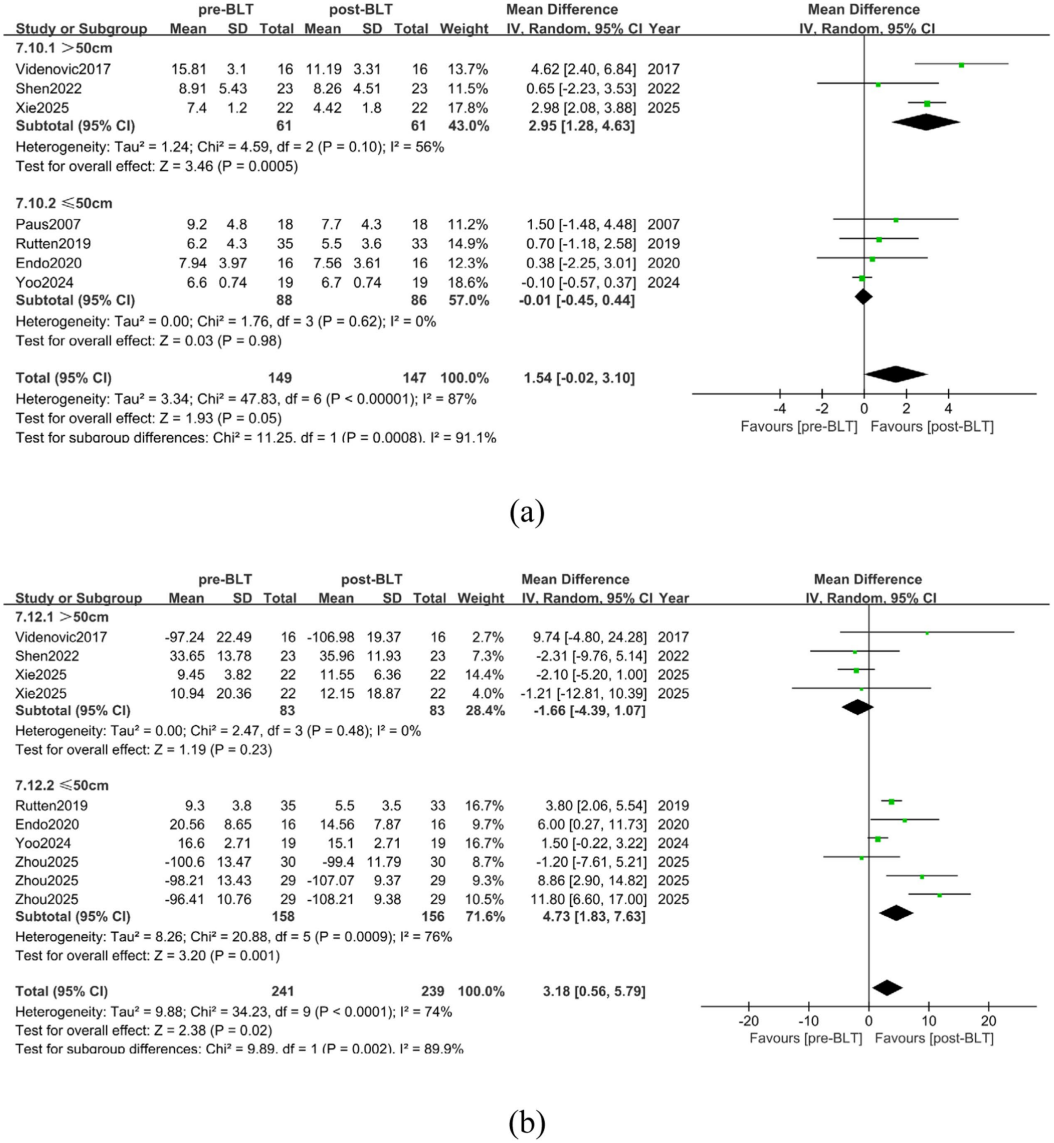
The influence of light source distance on the therapeutic effect of BLT. **(a)** daytime sleepiness **(b)** nighttime sleep.

Five of the included trials placed the light source 50 cm distant from the patients (25, 28, 32, 34, 35). The distance parameters were not explicitly stated in two investigations (29, 31). Due to the different settings of the light source distance between the BLT group and the control group in some studies, in this subgroup analysis, only the comparison analysis between BLT and the baseline condition was conducted. The results of the meta-analysis showed that in alleviating the daytime sleepiness of PD patients, when the set light source distance was > 50 cm, the therapeutic effect of BLT tended to be after treatment (SMD = 2.95, 95% CI = [1.28, 4.63]), while when the distance was ≤ 50 cm, the therapeutic effect tended to be before treatment (SMD = -0.01, 95% CI = [−0.45, 0.44]), and the difference between the groups was statistically significant (*p* = 0.0008, SMD = 1.54, 95% CI = [−0.02, 3.10]), as shown in Figure 13a.

In improving the nighttime sleep of PD patients, when the light source distance was > 50 cm, the therapeutic effect of BLT tended to be before treatment (SMD = -1.66, 95% CI = [−4.39, 1.07]), and when the light source distance was ≤ 50 cm, the therapeutic effect tended to be after treatment (SMD = 4.73, 95% CI = [1.83, 7.63]), while at 50 cm, the difference between the two groups was statistically significant (*p* = 0.002, SMD = 3.18, 95% CI = [0.56, 5.79]), as shown in Figure 13b. However, the distance of the light source did not affect the therapeutic effects of BLT on depression (*p* = 0.08, 2.04 [−0.17, 4.25]), anxiety (*p* = 0.70, 0.65 [−0.32, 1.62]), cognition (*p* = 0.43, 0.16 [−0.85, 1.17]), fatigue (*p* = 0.78, 1.20 [−0.19, 2.58]), quality of life (*p* = 0.19, 0.04 [−0.36, 0.43]), sleep quality (*p* = 0.70, 0.65 [−0.32, 1.62]), and cognition (*p* = 0.43, 0.16 [−0.85, 1.17]) and fatigue (*p* = 0.78, 1.94 [1.29, 2.59]), as shown in Supplementary Figure 11.

#### 3.4.4 The intensity of BLT

The light intensity was divided into two groups based on the included studies: greater than 5,000 lux (25, 27, 28, 30, 31, 33, 34) and equal to or less than 5,000 lux (24, 26, 29, 32, 33, 35). The subgroup analysis only compared the intervention pre- and post- BLT because the intensity of bright light therapy varied amongst the control groups of various studies. The meta-analysis found that the intensity of BLT had no significant effect on the treatment effects of depression in Parkinson’s disease (*p* = 0.50, SMD = 0.38, 95% CI = [−0.01, 0.76]), anxiety (*p* = 0.89, SMD = 0.25, 95% CI = [−0.12, 0.62]), fatigue (*p* = 0.48, SMD = 0.36, 95% CI = [0.03, 0.69]), daytime sleepiness (*p* = 0.07, SMD = 0.48, 95% CI = [0.02, 0.94]) or nighttime sleep (*p* = 0.76, SMD = 0.39, 95% CI = [0.04, 0.74]), and quality of life (*p* = 0.66, SMD = 0.16, 95% CI = [−0.09, 0.40]), as shown in Supplementary Figure 12.

### 3.5 Adverse events

Seven studies reported on adverse events. One study recorded that no adverse events occurred with BLT treatment (29). BLT-related adverse events are usually transient and mild, and most disappear spontaneously or are gradually tolerated during the continuation of treatment. All recorded major adverse events (such as fractures caused by falls, transient cerebral ischemia episodes, etc.) were determined by the researchers to be unrelated to BLT treatment. Some neurological-related adverse events (such as dizziness and headache) were reported to occur more frequently in the BLT group, but the specific underlying mechanism is still unknown. Furthermore, the incidence of adverse events under various treatment frequencies did not differ statistically significantly (31).

### 3.6 Sensitivity analysis

**Figure 14:**
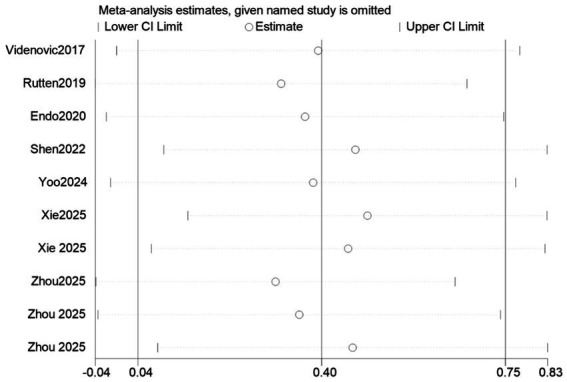
Sensitivity analysis.

When conducting a Meta-analysis of the improvement of nighttime sleep in PD patients by BLT, it was revealed that there was a high degree of heterogeneity, as seen in Figure 8b. To examine the robustness of the data, a sensitivity analysis was undertaken (by deleting each study one by one). The findings revealed that, while the direction of the total impact value remained positive, the statistical significance was relatively sensitive to the exclusion of specific research. Specifically, after eliminating Sonja et al. (30), Endo et al. (24), Yoo et al. (26) and certain data from Xiaoping (33), the confidence interval will cross the zero line (*p* > 0.05), losing statistical significance. Nevertheless, the general trend remained confirmed the beneficial effect of BLT on nighttime sleep. The final combined analysis results showed that BLT had a statistically significant effect on nighttime sleep (95% CI did not contain zero). The results of the sensitivity analysis are detailed in as shown in Table 4 and Figure 14.

**Table 4:** Sensitivity analysis.

Study omitted	Estimate	95% confidence interval
Videnovic et al. (2017) (28)	0.39134211	[0.00187661, 0.78080761]
Sonja et al. (2019) (30)	0.31974042	[−0.03941648, 0.67889733]
Endo et al. (2020) (24)	0.36583607	[−0.01821363, 0.74988576]
Yun et al. (2022) (25)	0.46314297	[0.0926566, 0.83362934]
Yoo et al. (2024) (26)	0.38154391	[−0.00983011, 0.77291793]
Xie et al. (2025) (34)	0.48640437	[0.13949675, 0.83331199]
Xie et al. (2025) (34)	0.44918323	[0.06880307, 0.8295634]
Xiaoping (2025) (33)	0.30885219	[−0.03815436, 0.65585873]
Xiaoping (2025) (33)	0.35474977	[−0.03427508, 0.74377461]
Xiaoping (2025) (33)	0.45779182	[0.08105446, 0.83452917]
Combined	0.39800145	[0.04300865, 0.75299425]

### 3.7 Publication bias

Publication bias was analyzed using Egger’s test across studies evaluating the effect of BLT on nighttime sleep in patients with PD. No significant publication bias was identified (*p* = 0.837), as shown in Table 5.

**Table 5:** Egger’s test.

Number of studies = 10					Root MSE = 2.028
Std_Eff	Coefficient	Std. err.	t	*P* > t	[95% conf. interval]
slope	0.759	1.71	0.44	0.669	[−3.184, 4.701]
bias	−1.243	5.858	−0.21	0.837	[−14.75,12.265]
Test of H0: no small-study effects			*P* = 0.837		

## 4 Discussion

### 4.1 Effect of BLT on non-motor symptoms in PD patients

#### 4.1.1 Depression

The meta-analysis’s findings demonstrate that BLT did not significantly improve PD patients’ depression when compared to the control group. This is consistent with the research results of Huang et al. (18). It should be noted that the subgroup evaluated using the HDRS scale in Sonja et al. (30) showed a major effect value. The studies’ heterogeneity was significantly reduced when this subgroup was eliminated and the overall analysis showed that BLT can effectively alleviate depression. This may be because the content evaluated by the HDRS scale has a relatively high proportion of physical symptoms, while the subgroup evaluated for depression using the GDS-30 scale in Sonja et al. (30) did not show significant abnormalities in the effect value. This scale focuses on emotional symptoms, so it is not impossible to assume that using the HDRS assessment method may exaggerate the treatment effect; in addition, considering the improvement of sleep disorders in patients by BLT in this study, we speculate that the therapeutic effect of BLT is more prominently reflected in the improvement of physical symptoms, while the alleviation of emotional symptoms is relatively slower (36).

On the other hand, the therapeutic effect may also be influenced by the differences in the BLT protocol. Sonja et al. (30) employed a higher frequency and a longer intervention period of BLT. A study using BLT to treat depression in adolescents showed that the duration of BLT affected the therapeutic effect (37). From a mechanistic perspective, the antidepressant effect of BLT depends on the regulation of the circadian rhythm system and monoamine neurotransmitters, and variations in clinical protocols may prevent these neurophysiological pathways from being fully activated (38). Therefore, we hypothesize that there may also be a “dose–response relationship” in the alleviation of patients’ depression by BLT. Most current studies use short-term and low-frequency treatment protocols, which may not be adequate to properly activate the necessary neurophysiological pathways, therefore resulting in discrepancies in the therapeutic impact of BLT.

#### 4.1.2 Anxiety

The meta-analysis results indicate that bright light therapy and dim light therapy do not significantly alleviate anxiety in people with PD. This result is consistent with the conclusion of Sun et al. (17). It is worth noting that in patients with seasonal anxiety disorder (39) and focal epilepsy (40), BLT has shown an improvement effect on anxiety symptoms. Research attention on anxiety symptoms in PD remains insufficient. However, anxiety is even more common in PD patients than depression (41), and it is closely related to the decline in quality of life and the increase in mortality (42). However, there are currently just a few specialized research on how BLT reduced anxiety in PD patients, and there are issues like variable light intensity settings and typically small sample numbers. The consistency and dependability of the outcomes could be impacted by each of these elements. About the anxiety-alleviating impact of BLT in the treatment of patients with PD, we are unable to reach a definitive judgment.

Therefore, it is necessary to conduct large-sample, strictly designed randomized controlled trials in the future. On the basis of unifying the lighting parameters and reasonably controlling the placebo effect, the intervention effect, and the mechanism of BLT on anxiety symptoms in PD patients, it should be further clarified.

#### 4.1.3 Cognition

The findings of this study demonstrate that BLT does not enhance the cognitive status of individuals with Parkinson’s disease, and dim light may worsen their cognitive abilities. Nonetheless, owing to the restricted quantity of research incorporated, this outcome should be approached with cautiousness. Studies have shown that BLT slows down the cognitive deterioration of dementia patients, while dim light cannot alleviate the cognitive decline (43). Strong light can activate non-visual photosensory pathways, regulate the orexin system, enhance hippocampal plasticity and the expression of hippocampal brain-derived neurotrophic factors, regulate the serotonin system, regulate biological rhythms and emotional states, and indirectly support cognitive processes. The Nile grass mouse has a high degree of diurnal activity. A research result shows that the task performance of the Nile grass mouse under strong light treatment is better and its spatial memory is stronger, while the spatial learning and memory abilities are impaired under dim light treatment (44). Therefore, the selection of BLT intensity should be rigorous to avoid cognitive impairment in PD patients caused by dim light.

#### 4.1.4 Fatigue

According to a meta-analysis of pre- and post-BLT data, BLT could significantly alleviate the fatigue symptoms of PD patients. The meta-analysis of pre- and post- control showed that dim light was unable to reduce fatigue symptoms. However, a comparison of BLT and the control group showed that BLT’s effectiveness in reducing patients’ fatigue symptoms was not better than that of the control group.

Parkinson’s disease patients have damage to their brainstem serotonergic nuclei and limbic system. Serotonin is directly related to emotions, motivation, sleep, and fatigue. The pathological changes of the limbic system mainly involve the limbic system-cortex circuit that regulates emotions, motivation, and alertness. Its dysfunction may lead to reduced energy and exacerbated fatigue (45). Light is a potent natural factor that can modulate the serotonin level in the human brain (46), which may be the reason why BLT can improve the fatigue symptoms of Parkinson’s disease patients (47).

Furthermore, research has shown that a BLT intensity of above 10,000 lux is more effective in reducing fatigue of cancer patients (46). In the meta-analysis, the only relevant study was that conducted by Raymackers et al. (29), which found that the intensity used in the BLT group was 472.7 lux. However, after eliminating this trial, the treatment effect of the BLT group was not superior to that of the control group (SMD = 0.18, 95% CI = [−0.09, 0.44]), and the BLT did not improve the fatigue symptoms of the patients (SMD = 0.31, 95% CI = [−0.06, 0.68]). Additionally, the studies have shown that the greatest relief in cancer-related fatigue occurs when the BLT intensity is between 1,000 and 5,000 lux (48). Although cancer-related fatigue is not equal to the fatigue of PD patients, it nevertheless shows that the light therapy intensity used for treating the fatigue of PD patients may also fall within a certain range. However, in the meta-analysis of BLT’s efficacy on reducing fatigue symptoms in PD patients, the number of original research included was rather modest, mostly consisting of small-sample trials. This has an impact on the combined results’ robustness and dependability to some degree. Large-scale, carefully planned randomized controlled trials are therefore desperately needed in the future to confirm its effectiveness and investigate its mode of action in greater detail, which will help to define the ideal BLT procedure.

#### 4.1.5 Sleep

The therapeutic effect of BLT in alleviating daytime sleepiness and nighttime sleep was better than that of dim light in the control group. The meta-analysis of pre- and post- control showed that the treatment of the control group could improve the sleep quality of PD patients.

BLT can regulate the function of the suprachiasmatic nucleus, change the circadian rhythm of peripheral clock genes, restore the rhythm control of melatonin secretion in the suprachiasmatic nucleus, and synchronize the circadian rhythm (24, 28), thereby consolidating the sleep–wake cycle. BLT also helps stimulate the arousal nucleus in the brainstem, generating neuroarousal and alleviating daytime sleepiness symptoms (28, 30). BLT can reduce the interference of high cortisol on sleep and improve the subjective sleep quality of patients with PD (30). However, the improvement of sleep-related symptoms cannot rule out the stable rhythm caused by participating in the study and regular wake-up and sleep times (30). Furthermore, it is impossible to overlook the impact of behavioral therapies and psychological expectations when assessing patients’ sleep quality. Therefore, the combination of certain physiological regulatory activities and non-specific psychological and behavioral components is probably what causes BLT to help sleep issues. The original study exhibited significant heterogeneity in terms of BLT parameters (such as intensity, light exposure time, and treatment course), assessment tools, and control group design. This to some extent affected the robustness of the combined results. Future studies should conduct large-scale, standardized clinical trials, adopt more objective sleep monitoring indicators, and explore patient characteristics (such as baseline circadian rhythm type and specific sleep disorder subtypes) that can predict the efficacy of BLT sleep therapy. These developments will help achieve personalized treatment.

#### 4.1.6 Quality of life

BLT failed to significantly improve the quality of life of PD patients, which is consistent with the findings of Sun et al. (17). This could be because BLT’s therapeutic effect has not yet reached the threshold for improving the overall quality of life assessment, which could be caused by the intensity, cycle, or duration of BLT; or it could be because PD patients suffer from a variety of motor and non-motor symptoms that limit daily life and social activities, and the assessment of quality of life is subjective, which greatly affects patients’ evaluation of their own quality of life. Previous study has confirmed that BLT significantly improved the quality of life of patients with post-stroke insomnia (13), suggesting that its effectiveness may vary depending on the condition. In order to better understand how BLT affects Parkinson’s disease patients’ quality of life, more study is required to lengthen the treatment cycle of BLT and create more sensitive and thorough assessment instruments.

### 4.2 Long-term effects of BLT on non-motor symptoms in PD

After the completion of BLT treatment, during the one-month and subsequent follow-ups (with the longest duration being six months), the effects of BLT in alleviating patients’ depression, nighttime sleep, sleep quality and quality of life remained. However, the persistent improved impact was not significantly superior to that of low light. Multiple studies have demonstrated that BLT has a lasting alleviating effect on patients’ depressive moods, sleep problems, and quality of life (49–51). Moreover, compared to static BLT, which has constant intensity and temperature, some researchers think that dynamic BLT, which changes with the sun’s changes throughout the day, including the intensity and temperature of light, is more effective at reducing patients’ symptoms and sustaining the therapeutic effect (51). Therefore, even if the current research can demonstrate that patients still have short-term symptom reduction after BLT treatment, more research is still needed to determine the treatment’s long-term maintenance effect. The BLT received by the PD patients included in the study was static BLT. In the future, it may be possible to consider adopting the dynamic BLT mode and prolonging the follow-up period to provide a more solid evidence-based basis for the long-term non-drug management of PD patients. Additionally, compared with the dim light of the control group, BLT does not have a significant advantage. Therefore, the parameters of BLT still need to be explored to achieve the optimal treatment effect.

### 4.3 Cycle of BLT

This study found BLT duration (whether≥1 month) had no significant impact on improving patients’ emotional, or sleep symptoms, suggesting therapeutic effect may not depend solely on duration. However, existing studies disagree on the BLT optimal intervention cycle: Zhou and Xiang found ≥1 week of intervention is needed for effect (33), while some suggest 6 to 8 weeks (52). This discrepancy may relate to study population, disease severity, and BLT parameters, indicating BLT intervention duration may require individualization rather than a fixed standard. There is currently no agreement on the length of the best BLT intervention; further high-quality research is required to examine people with various clinical features and develop customized treatment regimens.

### 4.4 Light source distance

There are currently no studies exploring whether the therapeutic efficacy of BLT is impacted by the light source’s distance. The distance between the patient and the light source has varied depending on the study. The results of the meta-analysis show that when this distance exceeds 50 cm, the effect of BLT in treating daytime sleepiness is better. However, when this distance is ≤ 50 cm, it is more conducive to improving the effect of BLT in treating PD patients’ nighttime sleep. This may be because the distance of the light source may affect the intensity of BLT (53). The intensity of BLT diminishes as the distance of the light source rises, thereby affecting the improvement of non-motor symptoms in patients by BLT. According to the intervention goal, an appropriate distance setting is needed to maximize the therapeutic effect of BLT on the non-motor symptoms of PD patients.

### 4.5 Intensity of BLT

The study’s findings show that the ideal light intensity for BLT in treating non-motor symptoms cannot be determined using a dividing point of 5,000 lux. However, BLT is more effective than the dim light in enhancing nighttime sleep compared to the control group. Therefore, the separating point between strong light and dim light intensity still needs to be further established; in addition, the distance of the light source is likely to be an essential element impacting the intensity of BLT, and future research should not overlook this.

Additionally, some researchers believe that BLT may trigger widespread neural activation and produce effects relatively quickly within a short period of time, while dim light therapy may require a longer period to achieve clinical improvement (34). According to a study, cancer patients’ sleep quality is improved more by BLT with an intensity of ≥ 5,000 lux than by those with an intensity of < 5,000 lux (54). According to a different study, patients’ sleep quality may be enhanced by BLT intensities between 1,000 and 10,000 lux (33). Therefore, we still need to conduct further research to determine the optimal light intensity. Furthermore, the lighting in the daily living environment could potentially influence the therapeutic effect of BLT. In the included studies, only Endo et al. (24) reduced the interference of environmental lighting by using blackout curtains. Given that natural sunlight can reach 10,000–30,000 lux, while typical household lighting is only 50–200 lux, this uncontrolled environmental lighting may significantly affect the treatment outcome (53). Therefore, future research should focus on standardizing treatment parameters, controlling environmental interference factors, and extending the follow-up time to better evaluate the efficacy and mechanism of BLT.

### 4.6 BLT may be a feasible option for non-pharmacological treatment of PD patients in a family setting

This study shows that the meta-analysis of pre- and post- BLT results indicates that BLT can significantly improve the fatigue, daytime sleepiness, nighttime sleep, and sleep quality of PD patients. The adverse events of BLT are relatively few, and the intervention forms are diverse, including light boxes (25), light tubes (35), glasses (26), etc. Moreover, home-based BLT does not affect the treatment effect, and the treatment compliance is high (55). PD patients are not extremely mobile. Home-based BLT treatment is more convenient for them, decreasing the problem of transportation for PD patients when they need to visit the hospital for treatment. Some studies have confirmed that home-based BLT can still alleviate the symptoms of PD (24), and it can be considered as a means of home treatment for PD patients. It should be noted that the intensity, cycle, frequency, and treatment time should be set reasonably according to the patient’s own symptoms, and the appropriate BLT tools should be selected. Attention should be paid to controlling environmental influencing factors and monitoring the patient’s treatment situation in a timely manner to help PD patients better manage their symptoms.

## 5 Limitations

This study included a total of 12 studies, among which 9 were randomized controlled trials and 3 were non-randomized controlled trials. The overall sample size was limited, and most of the studies were small-sample trials. This led to a reduced statistical test power for the meta-analysis, an increased risk of type II errors, and a limitation in the applicability of the research conclusions to a broader population with PD. The heterogeneity of the BLT intervention protocol might be the main reason for the high statistical heterogeneity of some outcome indicators. Although a random effects model was used and sensitivity analysis was conducted, the conclusion’s clinical generalizability was limited, and it was difficult to provide clear and operational guidance for clinical practice. The meta-analysis mainly evaluated based on patient self-assessment scales, lacking objective physiological indicators, which might introduce recall bias and placebo effect, thereby overestimating the true efficacy of BLT to a certain extent. The intervention protocols of the control groups in different studies varied significantly, including the distance of the light source and the intensity of BLT, which might artificially narrow the efficacy difference between the treatment group and the control group, leading to an underestimation of the efficacy of BLT treatment. The existing studies mostly focused on short-term treatment effects and lacked systematic long-term follow-up data, making it difficult to fully assess the long-term effect of BLT. Although attempts were made to conduct subgroup analyses on key parameters such as intervention period and light intensity, no consistent conclusions were reached. The studies included in the meta-analysis mostly did not control for environmental light factors, which affected the assessment of the efficacy of BLT treatment.

## 6 Conclusion

This study’s findings suggest that BLT may enhance non-motor symptoms (including depression, sleep problems, and fatigue) in individuals with PD, while causing fewer side effects. It might work as a method for patients with PD to control their symptoms at home. Nevertheless, because to the mainly small sample sizes of the included trials, we are unable to draw a definitive conclusion regarding the efficacy of BLT in addressing non-motor symptoms in individuals with PD. Nevertheless, due to the limitations of previous research, such as small sample size, high heterogeneity of the BLT protocol, and short follow-up duration, the efficacy (especially the long-term benefits) of BLT still needs to be further evaluated through large-scale, standardized clinical trials. Future study must concentrate on identifying the ideal treatment parameters, regulating environmental confounding variables, and identifying the neurobiological mechanisms to enhance the specific application of BLT in the management of PD.

## Data Availability

The original contributions presented in the study are included in the article/Supplementary material, further inquiries can be directed to the corresponding author.
