# Correction: The impact of bright light therapy on non-motor symptoms in patients with Parkinson's disease: a systematic review and meta-analysis

**DOI:** 10.3389/fneur.2026.1823587

**Published:** 2026-04-10

**Authors:** Junjie Geng, Hanli Yu, Liping Tan

**Affiliations:** 1 School of Nursing, Suzhou Medical College, Soochow University, Suzhou, Jiangsu, China; 2 Department of Nursing, The Second Affiliated Hospital of Soochow University, Suzhou, Jiangsu, China

**Keywords:** bright light therapy, meta-analysis, non-motor symptoms, Parkinson's disease, sleep

In the published article, affiliation 1 was erroneously given as “School of Nursing, Soochow Medical College, Soochow University, Suzhou, Jiangsu, China”. The correct affiliation appears below:

“School of Nursing, Suzhou Medical College, Soochow University, Suzhou, Jiangsu, China”

In the published article no funding was declared. The updated funding statement appears below.

“The author(s) declared that financial support was received for this work and/or its publication. Open access funding was covered by Suzhou Medical Key Discipline (Clinical Nursing) Project Grant No. SZXK202102.”

The original version of this article has been updated.

