# Supplementary material for: The impact of bright light therapy on non-motor symptoms in patients with Parkinson’s disease: a systematic review and meta-analysis
Source: Front Neurol. 2026 Mar 3;17:1770673. doi: 10.3389/fneur.2026.1770673 (PMC12992288; doi:10.3389/fneur.2026.1770673)
Supplement: Supplementary file 1 [file Table_1.docx]

| Database | Search strategy |
| --- | --- |
| Embase | #1 'parkinson disease'/exp |
|  | #2 'idiopathic parkinsonism':ab,ti OR 'lewy bodies of parkinson disease':ab,ti OR 'lewy bodies of parkinson`s disease':ab,ti OR 'lewy bodies of parkinsons disease':ab,ti OR 'lewy body parkinson disease':ab,ti OR 'lewy body parkinson`s disease':ab,ti OR 'lewy body parkinsons disease':ab,ti OR 'paralysis agitans':ab,ti OR 'parkinson dementia complex':ab,ti OR 'parkinson`s disease':ab,ti OR 'parkinsons disease':ab,ti OR 'primary parkinsonism':ab,ti OR 'parkinson disease':ab,ti OR 'parkinson*':ab,ti OR 'parkinsonism':ab,ti OR 'parkinsonian syndrome':ab,ti OR 'pd':ab,ti OR 'lewy body disease':ab,ti |
|  | #3 'phototherapy'/exp |
|  | #4 'phototherapies':ab,ti OR 'therapy, photoradiation':ab,ti OR 'photoradiation therapies':ab,ti OR 'therapies, photoradiation':ab,ti OR 'light therapy':ab,ti OR 'light therapies':ab,ti OR 'therapies, light':ab,ti OR 'therapy, light':ab,ti OR 'heliotherapy':ab,ti OR 'light':ab,ti OR 'bright light':ab,ti OR 'sunlight':ab,ti OR 'artificial light':ab,ti OR 'phototherapy':ab,ti OR 'photoradiation therapy':ab,ti OR 'light exposure':ab,ti OR 'or light treatment':ab,ti OR 'blue light':ab,ti OR 'white light':ab,ti OR 'light box':ab,ti OR 'phototherapy effect':ab,ti OR 'continuous phototherapy':ab,ti OR 'intermittent phototherapy':ab,ti |
|  | #5 #1 OR #2 |
|  | #6 #3 OR #4 |
|  | #7 #5 AND #6 |
| Cochrane library | #1 [Parkinson Disease] explode all trees |
|  | #2 (“idiopathic parkinsonism”):ti,ab,kw OR (“Lewy bodies of Parkinson disease”):ti,ab,kw OR (“Lewy bodies of Parkinson`s disease'”):ti,ab,kw OR (“Lewy bodies of Parkinsons disease'”):ti,ab,kw OR (“Lewy body Parkinson disease”):ti,ab,kw OR (“Lewy body Parkinson`s disease”):ti,ab,kw OR (“Lewy body Parkinsons disease”):ti,ab,kw OR (“paralysis agitans”):ti,ab,kw OR (“Parkinson dementia complex”):ti,ab,kw OR (“Parkinson`s disease”):ti,ab,kw OR (“Parkinsons disease”):ti,ab,kw OR (“primary parkinsonism”):ti,ab,kw OR (“Parkinson disease”):ti,ab,kw OR (“parkinsonism”):ti,ab,kw OR (“parkinsonian syndrome”):ti,ab,kw OR (“PD”):ti,ab,kw OR (“lewy body disease”):ti,ab,kw OR ("'Parkinson dementia complex"):ti,ab,kw |
|  | #3 [Phototherapy] explode all trees |
|  | #4 (Phototherapies or Therapy, Photoradiation or Photoradiation Therapies or Therapies, Photoradiation or Light Therapy or Light Therapies or Therapies, Light or Therapy, Light or Photoradiation Therapy or heliotherapy or light or bright light or sunlight or artificial light or phototherapy or photoradiation therapy or blue light OR white light OR light exposure OR light box OR light treatment or Phototherapy effect or Continuous phototherapy or Intermittent phototherapy):ti,ab,kw |
|  | #5 #1 OR #2 |
|  | #6 #3 OR #4 |
|  | #7 #5 AND #6 |
| Pubmed | #1 "Parkinson Disease"[Mesh] |
|  | #2 (((((((((((((((((idiopathic parkinsonism[Title/Abstract]) OR (Lewy bodies of Parkinson disease[Title/Abstract])) OR (Lewy bodies of Parkinson`s disease[Title/Abstract])) OR (Lewy bodies of Parkinsons disease[Title/Abstract])) OR (Lewy body Parkinson disease[Title/Abstract])) OR (Lewy body Parkinson`s disease[Title/Abstract])) OR (Lewy body Parkinsons disease[Title/Abstract])) OR (paralysis agitans[Title/Abstract])) OR (Parkinson dementia complex[Title/Abstract])) OR (Parkinson`s disease[Title/Abstract])) OR (Parkinsons disease[Title/Abstract])) OR (primary parkinsonism[Title/Abstract])) OR (Parkinson disease[Title/Abstract])) OR (parkinsonism[Title/Abstract])) OR (parkinsonian syndrome[Title/Abstract])) OR (PD[Title/Abstract])) OR (lewy body disease[Title/Abstract])) OR (Parkinson dementia complex[Title/Abstract]) |
|  | #3 "Phototherapy"[Mesh] |
|  | #4 ((((((((((((((((((((Phototherapies[Title/Abstract]) OR (Therapy, Photoradiation[Title/Abstract])) OR (Photoradiation Therapies[Title/Abstract])) OR (Therapies, Photoradiation[Title/Abstract])) OR (Light Therapy[Title/Abstract])) OR (Light Therapies[Title/Abstract])) OR (Therapies, Light[Title/Abstract])) OR (Therapy, Light[Title/Abstract])) OR (Photoradiation Therapy[Title/Abstract])) OR (heliotherapy[Title/Abstract])) OR (light[Title/Abstract])) OR (bright light[Title/Abstract])) OR (sunlight[Title/Abstract])) OR (artificial light[Title/Abstract])) OR (phototherapy[Title/Abstract])) OR (photoradiation therapy[Title/Abstract])) OR (light exposure[Title/Abstract])) OR (light treatment[Title/Abstract])) OR (blue light[Title/Abstract])) OR (white light[Title/Abstract])) OR (light box[Title/Abstract]) OR (Phototherapy effect[Title/Abstract]) OR (Continuous phototherapy[Title/Abstract]) OR (Intermittent phototherapy[Title/Abstract]) |
|  | #5 ("Parkinson Disease"[Mesh]) OR ((((((((((((((((((idiopathic parkinsonism[Title/Abstract]) OR (Lewy bodies of Parkinson disease[Title/Abstract])) OR (Lewy bodies of Parkinson`s disease[Title/Abstract])) OR (Lewy bodies of Parkinsons disease[Title/Abstract])) OR (Lewy body Parkinson disease[Title/Abstract])) OR (Lewy body Parkinson`s disease[Title/Abstract])) OR (Lewy body Parkinsons disease[Title/Abstract])) OR (paralysis agitans[Title/Abstract])) OR (Parkinson dementia complex[Title/Abstract])) OR (Parkinson`s disease[Title/Abstract])) OR (Parkinsons disease[Title/Abstract])) OR (primary parkinsonism[Title/Abstract])) OR (Parkinson disease[Title/Abstract])) OR (parkinsonism[Title/Abstract])) OR (parkinsonian syndrome[Title/Abstract])) OR (PD[Title/Abstract])) OR (lewy body disease[Title/Abstract])) OR (Parkinson dementia complex[Title/Abstract])) |
|  | #6 ("Phototherapy"[Mesh]) OR (((((((((((((((((((((Phototherapies[Title/Abstract]) OR (Therapy, Photoradiation[Title/Abstract])) OR (Photoradiation Therapies[Title/Abstract])) OR (Therapies, Photoradiation[Title/Abstract])) OR (Light Therapy[Title/Abstract])) OR (Light Therapies[Title/Abstract])) OR (Therapies, Light[Title/Abstract])) OR (Therapy, Light[Title/Abstract])) OR (Photoradiation Therapy[Title/Abstract])) OR (heliotherapy[Title/Abstract])) OR (light[Title/Abstract])) OR (bright light[Title/Abstract])) OR (sunlight[Title/Abstract])) OR (artificial light[Title/Abstract])) OR (phototherapy[Title/Abstract])) OR (photoradiation therapy[Title/Abstract])) OR (light exposure[Title/Abstract])) OR (light treatment[Title/Abstract])) OR (blue light[Title/Abstract])) OR (white light[Title/Abstract])) OR (light box[Title/Abstract]) OR (Phototherapy effect[Title/Abstract]) OR (Continuous phototherapy[Title/Abstract]) OR (Intermittent phototherapy[Title/Abstract])) |
|  | #7 (("Parkinson Disease"[Mesh]) OR ((((((((((((((((((idiopathic parkinsonism[Title/Abstract]) OR (Lewy bodies of Parkinson disease[Title/Abstract])) OR (Lewy bodies of Parkinson`s disease[Title/Abstract])) OR (Lewy bodies of Parkinsons disease[Title/Abstract])) OR (Lewy body Parkinson disease[Title/Abstract])) OR (Lewy body Parkinson`s disease[Title/Abstract])) OR (Lewy body Parkinsons disease[Title/Abstract])) OR (paralysis agitans[Title/Abstract])) OR (Parkinson dementia complex[Title/Abstract])) OR (Parkinson`s disease[Title/Abstract])) OR (Parkinsons disease[Title/Abstract])) OR (primary parkinsonism[Title/Abstract])) OR (Parkinson disease[Title/Abstract])) OR (parkinsonism[Title/Abstract])) OR (parkinsonian syndrome[Title/Abstract])) OR (PD[Title/Abstract])) OR (lewy body disease[Title/Abstract])) OR (Parkinson dementia complex[Title/Abstract]))) AND (("Phototherapy"[Mesh]) OR (((((((((((((((((((((Phototherapies[Title/Abstract]) OR (Therapy, Photoradiation[Title/Abstract])) OR (Photoradiation Therapies[Title/Abstract])) OR (Therapies, Photoradiation[Title/Abstract])) OR (Light Therapy[Title/Abstract])) OR (Light Therapies[Title/Abstract])) OR (Therapies, Light[Title/Abstract])) OR (Therapy, Light[Title/Abstract])) OR (Photoradiation Therapy[Title/Abstract])) OR (heliotherapy[Title/Abstract])) OR (light[Title/Abstract])) OR (bright light[Title/Abstract])) OR (sunlight[Title/Abstract])) OR (artificial light[Title/Abstract])) OR (phototherapy[Title/Abstract])) OR (photoradiation therapy[Title/Abstract])) OR (light exposure[Title/Abstract])) OR (light treatment[Title/Abstract])) OR (blue light[Title/Abstract])) OR (white light[Title/Abstract])) OR (light box[Title/Abstract]) OR (Phototherapy effect[Title/Abstract]) OR (Continuous phototherapy[Title/Abstract]) OR (Intermittent phototherapy[Title/Abstract]))) |
| Web of Science | #1 (((((((((((((((((((((((((AB=(Phototherapy)) OR AB=(Phototherapies)) OR AB=(Therapy, Photoradiation)) OR AB=(Photoradiation Therapies)) OR AB=(Therapies, Photoradiation)) OR AB=(Light Therapy)) OR AB=(Light Therapies)) OR AB=(Therapies, Light )) OR AB=(Therapy, Light)) OR AB=(Photoradiation Therapy)) OR AB=(heliotherapy)) OR AB=(light)) OR AB=(bright light)) OR AB=(sunlight))) OR AB=(artificial light)) OR AB=(phototherapy)) OR AB=(photoradiation therapy)) OR AB=(blue light)) OR AB=(white light)) OR AB=(light exposure)) OR AB=(light box)) OR AB=(light treatment)) OR AB=(Phototherapy effect)) OR AB=(Continuous phototherapy)) OR AB=(Intermittent phototherapy) |
|  | #2 (((((((((((((((((AB=(idiopathic parkinsonism)) OR AB=(Lewy bodies of Parkinson disease)) OR AB=(Lewy bodies of Parkinson`s disease)) OR AB=(Lewy bodies of Parkinsons disease)) OR AB=(Lewy body Parkinson disease)) OR AB=(Lewy body Parkinson`s disease)) OR AB=(Lewy body Parkinsons disease)) OR AB=(paralysis agitans)) OR AB=(Parkinson dementia complex)) OR AB=(Parkinson`s disease)) OR AB=(Parkinsons disease)) OR AB=(primary parkinsonism)) OR AB=(Parkinson disease)) OR AB=(parkinsonism)) OR AB=(PD)) OR AB=(parkinsonian syndrome)) OR AB=(lewy body disease)) OR AB=(Parkinson dementia complex) |
|  | #3 #1 AND #2 |
| OVID-Medline | #1 Parkinson Disease/ |
|  | #2 (idiopathic parkinsonism or Lewy bodies of Parkinson disease or Lewy bodies of Parkinson`s disease or Lewy bodies of Parkinsons disease or Lewy body Parkinson disease or Lewy body Parkinson`s disease or Lewy body Parkinsons disease or paralysis agitans or Parkinson dementia complex or Parkinson`s disease or Parkinsons disease or primary parkinsonism or Parkinson disease or parkinson* or parkinsonism or parkinsonian syndrome or PD or lewy body disease).ab,ti,kw. |
|  | #3 Phototherapy/ |
|  | #4 (Phototherapies or Therapy, Photoradiation or Photoradiation Therapies or Therapies, Photoradiation or Light Therapy or Light Therapies or Therapies, Light or Therapy, Light or Photoradiation Therapy or heliotherapy or light or bright light or sunlight or artificial light or phototherapy or photoradiation therapy or light exposure or light treatment or blue light or white light or light box or Phototherapy effect or Continuous phototherapy or Intermittent phototherapy).ab,ti,kw. |
|  | #5 #1 OR #2 |
|  | #6 #3 OR #4 |
|  | #7 #5 AND #6 |
| China knowledge network database (CNKI) | (( TKA%= 'Parkinson's disease' OR SU%='Parkinson's disease') OR (TKA%= 'parkinson disease' OR SU%='parkinson disease') OR (TKA%= 'parkinsonian' OR SU%='parkinsonian') OR (TKA %= 'paralysis agitans' OR SU%='paralysis agitans') OR (TKA%= 'Parkinsonism' OR SU%='Parkinsonism') OR (TKA%= 'Parkinson' OR SU%='Parkinson')) AND ((TKA%= 'Phototherapy' OR SU%='Phototherapy' ) OR (TKA%= 'light therapeutic' OR SU%='light therapeutic') OR (TKA%= 'Phototherapy treatment' OR SU%='Phototherapy treatment') OR (TKA%= 'bright light therapy' OR SU%='bright light therapy') OR (TKA%= 'Blue light therapy' OR SU%='Blue light therapy') OR (TKA%= 'Intermittent bright light therapy' OR SU%='Intermittent bright light therapy') OR (TKA%= 'Continuous bright light therapy' OR SU%='Continuous bright light therapy') OR (TKA%= 'Red light therapy' OR SU%='Red light therapy')) |
| Wanfang | subject:(Parkinson's disease OR parkinson disease OR parkinsonian OR paralysis agitans OR Lewy body Parkinson's disease OR Parkinsonism OR Parkinson) and subject:(light therapeutic OR Phototherapy OR bright light therapy OR Phototherapy treatment OR Blue light therapy OR Intermittent bright light therapy OR Continuous bright light therapy OR Red light therapy) |
| VIP | M=(Parkinson's disease OR parkinson disease OR parkinsonian OR paralysis agitans OR Lewy body Parkinson's disease OR Parkinsonism OR Parkinson) AND M=(light therapeutic OR Phototherapy OR bright light therapy OR Phototherapy treatment OR Blue light therapy OR Intermittent bright light therapy OR Continuous bright light therapy OR Red light therapy) |
| Chinese biomedical database (CBM) | #1 "Parkinson Disease" [unweighted: expanded] |
|  | #2 "Parkinson's disease" [Common Fields:Intelligent] OR "parkinson disease" [Common Fields:Intelligent] OR "parkinsonian" [Common Fields:Intelligent] OR "paralysis agitans" [Common Fields:Intelligent] OR " Lewy body Parkinson's disease" [Common Fields:Intelligent] OR "Parkinson" [Common Fields:Intelligent] OR "Parkinsonism" [Common Fields:Intelligent] |
|  | #3 "Photochemotherapy" [unweighted: extended] |
|  | #4 "light therapeutic " [Common Fields:Intelligent] OR "Phototherapy" [Common Fields:Intelligent] OR "bright light therapy" [Common Fields:Intelligent] OR "Phototherapy treatment" [Common Fields:Intelligent] OR "Blue light therapy" [Common Fields:Intelligent] OR "Intermittent bright light therapy " [Common Fields:Intelligent] OR "Continuous bright light therapy" [Common Fields:Intelligent] OR"Red light therapy" [Common Fields:Intelligent] |
|  | #5 #1 OR #2 |
|  | #6 #3 OR #4 |
|  | #7 #5 AND #6 |
