# Supplementary material for: The impact of bright light therapy on non-motor symptoms in patients with Parkinson’s disease: a systematic review and meta-analysis
Source: Front Neurol. 2026 Mar 3;17:1770673. doi: 10.3389/fneur.2026.1770673 (PMC12992288; doi:10.3389/fneur.2026.1770673)
Supplement: Supplementary file 2 [file Supplementary_file_1.pdf]

Study or Subgroup	mean change in pre and post -control light group			mean change in pre and post - BLT group				Std. Mean Difference
	Mean	SD	Total	Mean	SD	Total	Weight	IV, Fixed, 95% CI
Paus2007	-2.2	5.51	18	-2.2	5.67	18	12.4%	0.00 [-0.65, 0.65]
Raymackers2019	-0.18	2.67	16	-0.12	1.15	16	11.0%	-0.03 [-0.72, 0.66]
Rutten2019	-2.2	6.23	34	-4.2	6.55	33	22.7%	0.31 [-0.17, 0.79]
Rutten2019	-6.2	4.16	34	-7.1	4.5	33	22.9%	0.21 [-0.27, 0.69]
Videnovic2017	1.06	9.17	15	-0.43	4.15	16	10.6%	0.21 [-0.50, 0.91]
Willis2018	-1.28	8.17	9	-1.03	12.88	9	6.2%	-0.02 [-0.95, 0.90]
Xie2025	0.49	8.12	20	-0.05	7.67	22	14.4%	0.07 [-0.54, 0.67]
**Total (95% CI)**			**146**			**147**	**100.0%**	**0.14 [-0.09, 0.37]**

Heterogeneity: Chi² = 1.16, df = 6 (P = 0.98); I² = 0%
Test for overall effect: Z = 1.23 (P = 0.22)

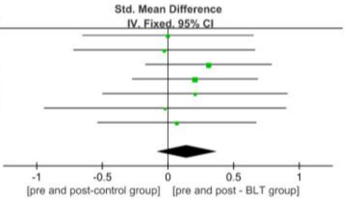

Supplementary Figure1

Study or Subgroup	pre-BLT			post-BLT				Std. Mean Difference	
	Mean	SD	Total	Mean	SD	Total	Weight	IV, Fixed, 95% CI	Year
Paus2007	9.9	6.1	18	7.7	5.1	18	11.4%	0.38 [-0.28, 1.04]	2007
Videnovic2017	8.31	3.63	16	7.88	4.53	16	10.3%	0.10 [-0.59, 0.80]	2017
Willis2018	8	13.99	9	6.97	11.37	9	5.8%	0.08 [-0.85, 1.00]	2018
Raymackers2019	5.31	1.15	16	5.19	1.15	16	10.3%	0.10 [-0.59, 0.80]	2019
Rutten2019	17.9	5.9	35	13.7	6.9	33	20.7%	0.65 [0.16, 1.14]	2019
Shen2022	7.96	4.25	23	8.52	4.03	23	14.8%	-0.13 [-0.71, 0.45]	2022
Yoo2024	10.6	1.56	20	10	1.56	20	12.6%	0.38 [-0.25, 1.00]	2024
Xie2025	5.84	8.06	22	5.79	7.21	22	14.2%	0.01 [-0.58, 0.60]	2025
**Total (95% CI)**			**159**			**157**	**100.0%**	**0.23 [0.01, 0.45]**	

Heterogeneity: Chi² = 5.66, df = 7 (P = 0.58); I² = 0%
Test for overall effect: Z = 2.05 (P = 0.04)

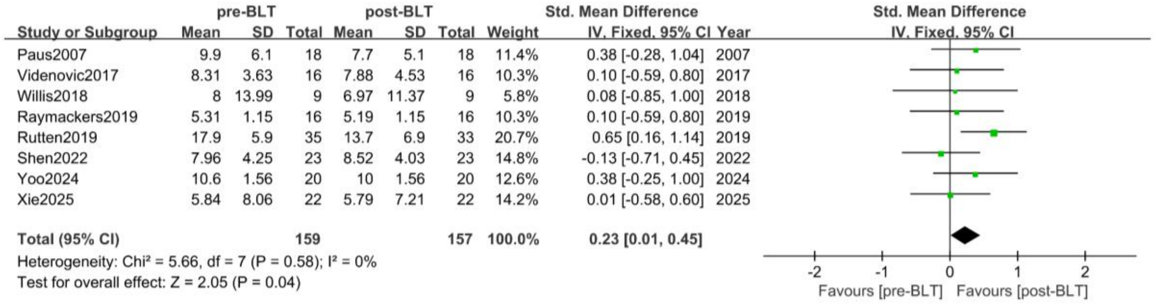

Supplementary Figure2

Study or Subgroup	pre-control			post-control				Std. Mean Difference	
	Mean	SD	Total	Mean	SD	Total	Weight	IV, Fixed, 95% CI	Year
Paus2007	8.7	6.1	18	6.5	1.5	18	15.5%	0.48 [-0.18, 1.15]	2007
Videnovic2017	8.27	4.71	15	9.33	10.57	15	13.3%	-0.13 [-0.84, 0.59]	2017
Willis2018	7.79	7.43	9	6.51	8.75	9	8.0%	0.15 [-0.78, 1.08]	2018
Rutten2019	17.1	5.9	37	14.9	6.3	34	31.1%	0.36 [-0.11, 0.83]	2019
Raymackers2019	5.81	2.67	16	5.63	2.7	16	14.3%	0.07 [-0.63, 0.76]	2019
Xie2025	6.02	8.91	20	6.51	7	20	17.8%	-0.06 [-0.68, 0.56]	2025
**Total (95% CI)**			**115**			**112**	**100.0%**	**0.18 [-0.08, 0.44]**	

Heterogeneity: Chi² = 2.74, df = 5 (P = 0.74); I² = 0%
Test for overall effect: Z = 1.35 (P = 0.18)

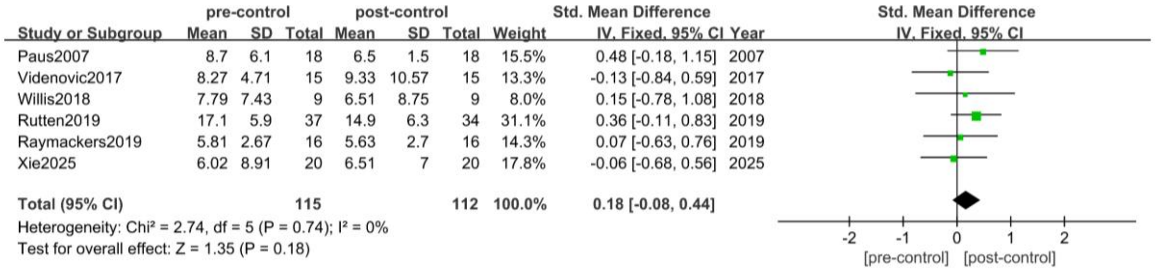

Supplementary Figure3

Study or Subgroup	mean change in pre and post -control light group			mean change in pre and post - BLT group				Std. Mean Difference	
	Mean	SD	Total	Mean	SD	Total	Weight	IV, Fixed, 95% CI	Year
Raymackers2019	-0.07	1.74	16	-0.25	1.59	16	43.3%	0.11 [-0.59, 0.80]	2019
Xie2025	-1.08	6.83	20	-1.36	6.26	22	56.7%	0.04 [-0.56, 0.65]	2025
**Total (95% CI)**			**36**			**38**	**100.0%**	**0.07 [-0.39, 0.53]**	

Heterogeneity: Chi² = 0.02, df = 1 (P = 0.89); I² = 0%
Test for overall effect: Z = 0.30 (P = 0.77)

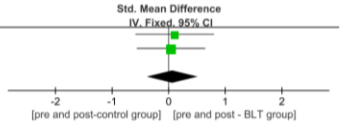

Supplementary Figure4

Study or Subgroup	mean change in pre and post -control light group			mean change in pre and post - BLT group				Std. Mean Difference	
	Mean	SD	Total	Mean	SD	Total	Weight	IV, Fixed, 95% CI	Year
Paus2007	-1.4	4.61	18	-1.5	4.57	18	17.6%	0.02 [-0.63, 0.67]	2007
Videnovic2017	-1.8	4.14	15	-4.62	3.21	16	14.1%	0.74 [0.01, 1.48]	2017
Rutten2019	-1.1	4.61	34	-0.7	4.05	33	32.8%	-0.09 [-0.57, 0.39]	2019
Raymackers2019	0.44	2.29	16	-0.44	2.29	16	15.4%	0.37 [-0.33, 1.07]	2019
Xie2025	-2.4	1.44	20	-2.98	1.59	22	20.1%	0.37 [-0.24, 0.99]	2025
**Total (95% CI)**			**103**			**105**	**100.0%**	**0.21 [-0.06, 0.49]**	

Heterogeneity: Chi² = 4.38, df = 4 (P = 0.36); I² = 9%
Test for overall effect: Z = 1.51 (P = 0.13)

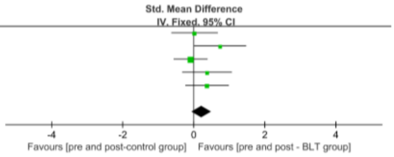

Supplementary Figure5

Study or Subgroup	pre-BLT Mean	pre-BLT SD	pre-BLT Total	post-BLT Mean	post-BLT SD	post-BLT Total	Weight	Std. Mean Difference IV, Fixed, 95% CI	Year
Paus2007	9.2	4.8	18	7.7	4.3	18	12.9%	0.32 [-0.34, 0.98]	2007
Videnovic2017	15.81	3.1	16	11.19	3.31	16	9.1%	1.40 [0.62, 2.19]	2017
Raymackers2019	9.06	2.29	16	8.62	2.29	16	11.5%	0.19 [-0.51, 0.88]	2019
Rutten2019	6.2	4.3	35	5.5	3.6	33	24.5%	0.17 [-0.30, 0.65]	2019
Endo2020	7.94	3.97	16	7.56	3.61	16	11.6%	0.10 [-0.60, 0.79]	2020
Shen2022	8.91	5.43	23	8.26	4.51	23	16.6%	0.13 [-0.45, 0.71]	2022
Yoo2024	6.6	0.74	19	6.7	0.74	19	13.7%	-0.13 [-0.77, 0.50]	2024
**Total (95% CI)**			**143**			**141**	**100.0%**	**0.25 [0.01, 0.48]**	

Heterogeneity: $Chi^2 = 10.24$, df = 6 (P = 0.12); $I^2 = 41\%$
Test for overall effect: Z = 2.05 (P = 0.04)

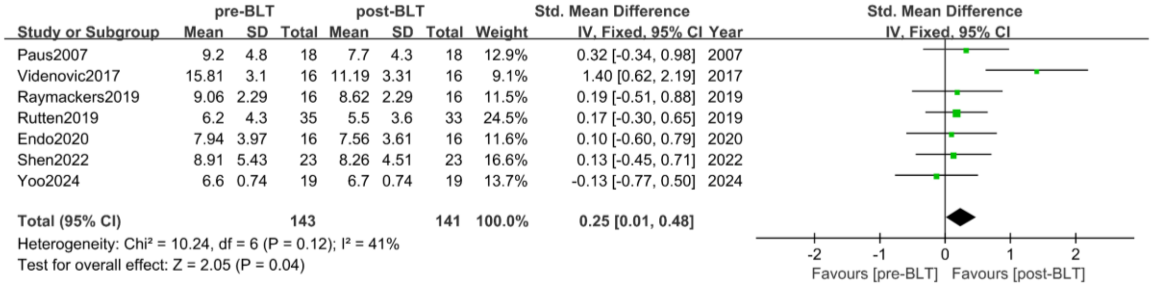

Supplementary Figure6

Study or Subgroup	pre-BLT Mean	pre-BLT SD	pre-BLT Total	post-BLT Mean	post-BLT SD	post-BLT Total	Weight	Std. Mean Difference IV, Fixed, 95% CI	Year
Videnovic2017	7.88	4.11	16	6.25	4.27	16	26.2%	0.38 [-0.32, 1.08]	2017
Shen2022	9.22	4.74	23	7.65	3.79	23	37.7%	0.36 [-0.22, 0.94]	2022
Xie2025	8.83	5.73	22	7	3.82	22	36.1%	0.37 [-0.23, 0.97]	2025
**Total (95% CI)**			**61**			**61**	**100.0%**	**0.37 [0.01, 0.73]**	

Heterogeneity: $Chi^2 = 0.00$, df = 2 (P = 1.00); $I^2 = 0\%$
Test for overall effect: Z = 2.01 (P = 0.04)

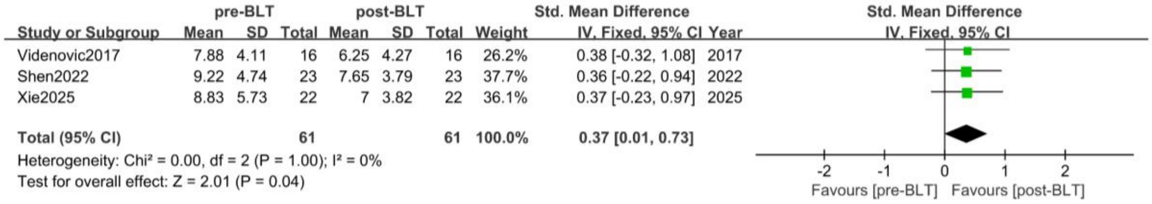

Supplementary Figure7

Study or Subgroup	mean change in pre and post -control light group Mean	SD	Total	mean change in pre and post - BLT group Mean	SD	Total	Weight	Std. Mean Difference IV, Fixed, 95% CI	Year
Paus2007	-0.4	4.81	18	-1.6	7.29	18	14.2%	0.19 [-0.46, 0.84]	2007
Videnovic2017	2.6	23.62	15	1.54	17.5	16	12.2%	0.05 [-0.65, 0.75]	2017
Willis2018	-1.12	3.42	9	-0.19	4.31	9	7.1%	-0.23 [-1.16, 0.70]	2018
Willis2018	0.19	0.76	9	0	0.44	9	7.0%	0.29 [-0.64, 1.22]	2018
Rutten2019	-2.5	12.09	34	-4.3	13.55	33	26.4%	0.14 [-0.34, 0.62]	2019
Xie2025	-0.08	5.74	20	0.14	5.34	22	16.6%	-0.04 [-0.64, 0.57]	2025
Xie2025	-3.72	23.45	20	-5.14	15.27	22	16.6%	0.07 [-0.53, 0.68]	2025
**Total (95% CI)**			**125**			**129**	**100.0%**	**0.08 [-0.17, 0.33]**	

Heterogeneity: $Chi^2 = 0.94$, df = 6 (P = 0.99); $I^2 = 0\%$
Test for overall effect: Z = 0.63 (P = 0.53)

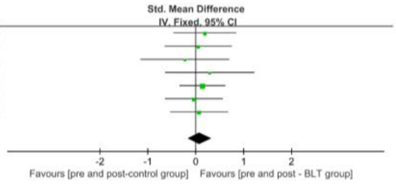

Supplementary Figure8

Subgroup analysis

Long-term effects of BLT on non-motor symptoms in PD

Supplementary Figure9

Study or Subgroup	pre-BLT Mean	pre-BLT SD	pre-BLT Total	post-BLT Mean	post-BLT SD	post-BLT Total	Weight	Std. Mean Difference IV, Random, 95% CI	Year
Videnovic2017	8.31	3.63	16	7.06	5.56	16	17.5%	0.26 [-0.44, 0.96]	2017
Rutten2019	14.7	3.5	35	7.6	5	33	20.1%	1.63 [1.08, 2.19]	2019
Rutten2019	17.9	5.9	35	13.7	6.9	33	21.3%	0.65 [0.16, 1.14]	2019
Rutten2019	17.9	5.9	35	13.2	6.6	30	21.0%	0.75 [0.24, 1.25]	2019
Rutten2019	14.7	3.5	35	8.5	4.6	30	20.1%	1.51 [0.96, 2.07]	2019
**Total (95% CI)**			**156**			**142**	**100.0%**	**0.97 [0.48, 1.46]**	

Heterogeneity: Tau² = 0.23; Chi² = 15.64, df = 4 (P = 0.004); I² = 74%
Test for overall effect: Z = 3.90 (P < 0.0001)

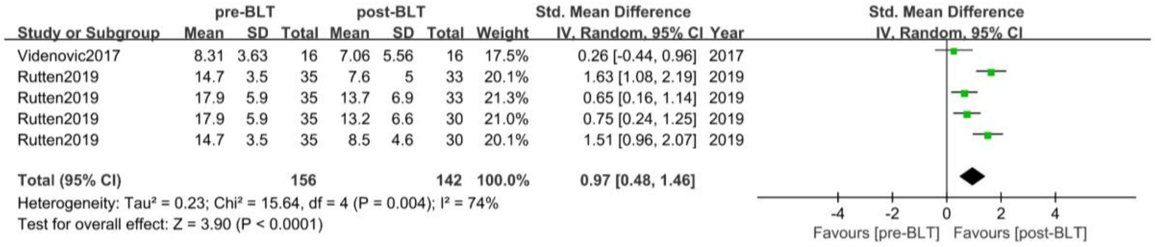

(a)Depression

Study or Subgroup	pre-BLT Mean	pre-BLT SD	pre-BLT Total	post-BLT Mean	post-BLT SD	post-BLT Total	Weight	Std. Mean Difference IV, Fixed, 95% CI	Year
Videnovic2017	-97.24	22.49	16	-103.23	21.76	16	21.2%	0.26 [-0.43, 0.96]	2017
Rutten2019	9.3	3.8	35	5.5	3.5	33	39.8%	1.03 [0.52, 1.53]	2019
Rutten2019	9.3	3.8	35	6.1	3.2	30	39.0%	0.89 [0.38, 1.41]	2019
**Total (95% CI)**			**86**			**79**	**100.0%**	**0.81 [0.49, 1.13]**	

Heterogeneity: Chi² = 3.17, df = 2 (P = 0.21); I² = 37%
Test for overall effect: Z = 4.98 (P < 0.00001)

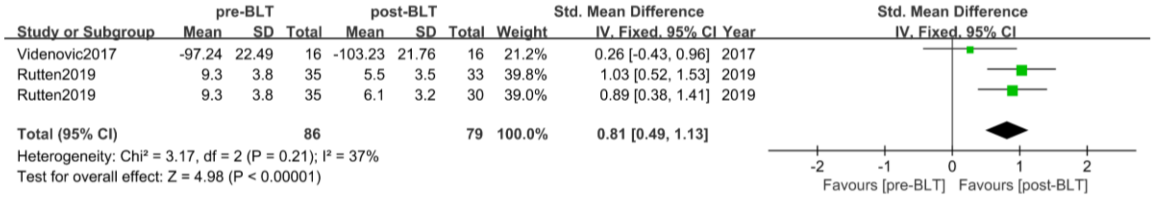

(b)Nighttime sleep

Study or Subgroup	pre-BLT Mean	pre-BLT SD	pre-BLT Total	post-BLT Mean	post-BLT SD	post-BLT Total	Weight	Std. Mean Difference IV, Fixed, 95% CI	Year
Videnovic2017	7.88	4.11	16	5.56	3.6	16	21.3%	0.59 [-0.12, 1.30]	2017
Rutten2019	4.3	1.5	35	2.3	1.5	33	38.6%	1.32 [0.79, 1.85]	2019
Rutten2019	4.3	1.5	35	2.8	1.5	30	40.0%	0.99 [0.47, 1.51]	2019
**Total (95% CI)**			**86**			**79**	**100.0%**	**1.03 [0.70, 1.36]**	

Heterogeneity: Chi² = 2.68, df = 2 (P = 0.26); I² = 25%
Test for overall effect: Z = 6.16 (P < 0.00001)

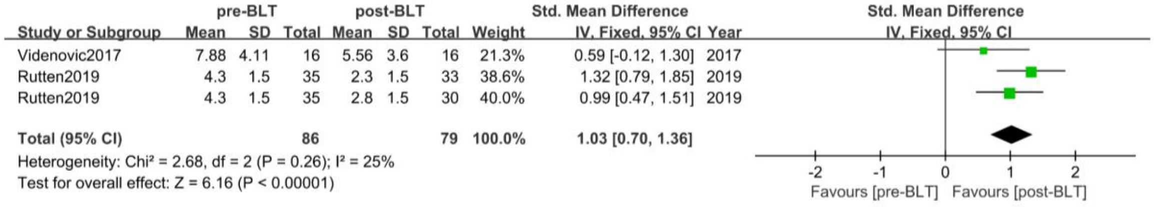

(c)Sleep quality

Study or Subgroup	pre-BLT Mean	pre-BLT SD	pre-BLT Total	post-BLT Mean	post-BLT SD	post-BLT Total	Weight	Std. Mean Difference IV, Fixed, 95% CI	Year
Videnovic2017	41.46	19.3	16	41	18.68	16	19.8%	0.02 [-0.67, 0.72]	2017
Rutten2019	-79	13.1	35	-83.3	13.6	33	41.5%	0.32 [-0.16, 0.80]	2019
Rutten2019	-79	13.1	35	-85.5	12.6	30	38.7%	0.50 [0.00, 0.99]	2019
**Total (95% CI)**			**86**			**79**	**100.0%**	**0.33 [0.02, 0.64]**	

Heterogeneity: Chi² = 1.20, df = 2 (P = 0.55); I² = 0%
Test for overall effect: Z = 2.10 (P = 0.04)

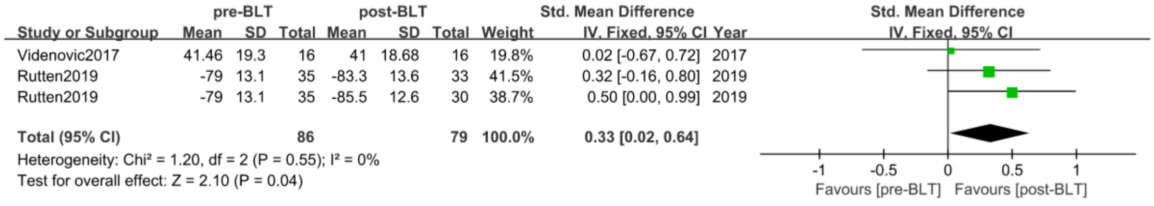

(d)Quality of life

Study or Subgroup	pre-BLT Mean	pre-BLT SD	pre-BLT Total	post-BLT Mean	post-BLT SD	post-BLT Total	Weight	Std. Mean Difference IV, Random, 95% CI	Year
Videnovic2017	15.81	3.1	16	11.38	4.15	16	25.4%	1.18 [0.42, 1.94]	2017
Rutten2019	6.2	4.3	35	5.1	3.8	30	37.0%	0.27 [-0.22, 0.76]	2019
Rutten2019	6.2	4.3	35	5.5	3.6	33	37.6%	0.17 [-0.30, 0.65]	2019
**Total (95% CI)**			**86**			**79**	**100.0%**	**0.46 [-0.06, 0.98]**	

Heterogeneity: Tau² = 0.13; Chi² = 5.19, df = 2 (P = 0.07); I² = 61%
Test for overall effect: Z = 1.74 (P = 0.08)

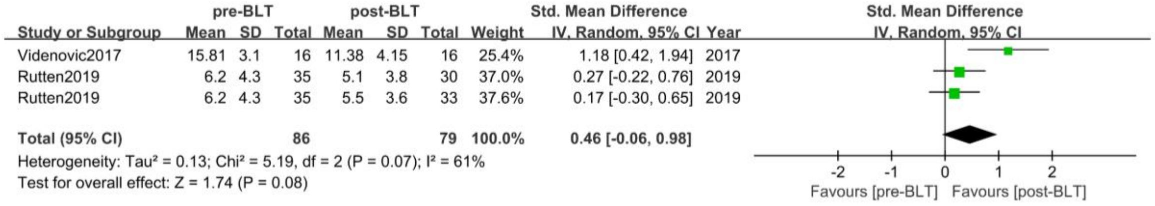

(e)Daytime sleepiness

Supplementary Figure9 Long-term effects of BLT on non-motor symptoms in PD

Supplementary Figure10

Study or Subgroup	pre BLT Mean	pre BLT SD	pre BLT Total	post BLT Mean	post BLT SD	post BLT Total	Weight	Std. Mean Difference IV, Random, 95% CI	Year
**6.2.1 ≥1month**									
Raymackers2019	5.31	1.15	16	5.19	1.15	16	10.6%	0.10 [-0.59, 0.80]	2019
Xie2025	5.84	8.06	22	5.79	7.21	22	11.7%	0.01 [-0.58, 0.60]	2025
**Subtotal (95% CI)**			**38**			**38**	**22.2%**	**0.05 [-0.40, 0.50]**	
Heterogeneity: Tau² = 0.00; Chi² = 0.04, df = 1 (P = 0.84); I² = 0%									
Test for overall effect: Z = 0.20 (P = 0.84)									
**6.2.2 <1month**									
Paus2007	9.9	6.1	18	7.7	5.1	18	10.9%	0.38 [-0.28, 1.04]	2007
Videnovic2017	8.31	3.63	16	7.88	4.53	16	10.6%	0.10 [-0.59, 0.80]	2017
Willis2018	8	13.99	9	6.97	11.37	9	8.3%	0.08 [-0.85, 1.00]	2018
Rutten2019	14.7	3.5	35	7.6	5	33	12.1%	1.63 [1.08, 2.19]	2019
Rutten2019	17.9	5.9	35	13.7	6.9	33	12.8%	0.65 [0.16, 1.14]	2019
Shen2022	7.96	4.25	23	8.52	4.03	23	11.8%	-0.13 [-0.71, 0.45]	2022
Yoo2024	10.6	1.56	20	10	1.56	20	11.3%	0.38 [-0.25, 1.00]	2024
**Subtotal (95% CI)**			**156**			**152**	**77.8%**	**0.47 [-0.00, 0.93]**	
Heterogeneity: Tau² = 0.29; Chi² = 23.38, df = 6 (P = 0.0007); I² = 74%									
Test for overall effect: Z = 1.96 (P = 0.05)									
**Total (95% CI)**			**194**			**190**	**100.0%**	**0.38 [-0.01, 0.76]**	
Heterogeneity: Tau² = 0.24; Chi² = 26.91, df = 8 (P = 0.0007); I² = 70%									
Test for overall effect: Z = 1.93 (P = 0.05)									
Test for subgroup differences: Chi² = 1.62, df = 1 (P = 0.20), I² = 38.1%									

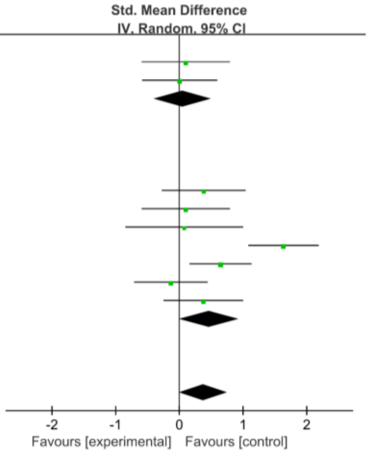

(a)Depression

Study or Subgroup	pre BLT Mean	pre BLT SD	pre BLT Total	post BLT Mean	post BLT SD	post BLT Total	Weight	Std. Mean Difference IV, Fixed, 95% CI
**6.3.1 ≥1month**								
Raymackers2019	7.75	1.59	16	7.5	1.59	16	28.3%	0.15 [-0.54, 0.85]
Xie2025	5.82	6.36	22	4.46	6.15	20	36.9%	0.21 [-0.39, 0.82]
**Subtotal (95% CI)**			**38**			**36**	**65.2%**	**0.19 [-0.27, 0.64]**
Heterogeneity: Chi² = 0.02, df = 1 (P = 0.90); I² = 0%								
Test for overall effect: Z = 0.80 (P = 0.42)								
**6.3.2 <1month**								
Yoo2024	10.6	1.62	20	10	1.62	20	34.8%	0.36 [-0.26, 0.99]
**Subtotal (95% CI)**			**20**			**20**	**34.8%**	**0.36 [-0.26, 0.99]**
Heterogeneity: Not applicable								
Test for overall effect: Z = 1.14 (P = 0.26)								
**Total (95% CI)**			**58**			**56**	**100.0%**	**0.25 [-0.12, 0.62]**
Heterogeneity: Chi² = 0.21, df = 2 (P = 0.90); I² = 0%								
Test for overall effect: Z = 1.32 (P = 0.19)								
Test for subgroup differences: Chi² = 0.20, df = 1 (P = 0.66), I² = 0%								

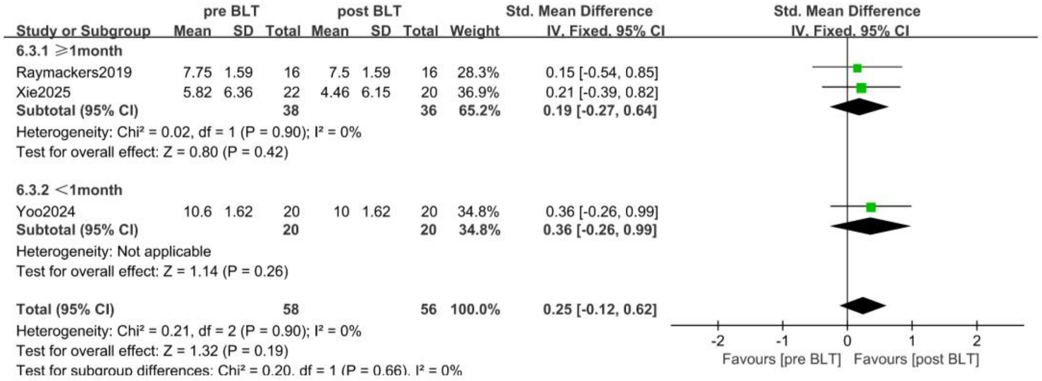

(b)Anxiety

Study or Subgroup	pre BLT Mean	pre BLT SD	pre BLT Total	post BLT Mean	post BLT SD	post BLT Total	Weight	Std. Mean Difference IV, Fixed, 95% CI	Year
**6.5.1 ≥1month**									
Xie2025	-25.55	5.51	22	-26.41	4.45	22	27.8%	0.17 [-0.42, 0.76]	2025
**Subtotal (95% CI)**			**22**			**22**	**27.8%**	**0.17 [-0.42, 0.76]**	
Heterogeneity: Not applicable									
Test for overall effect: Z = 0.56 (P = 0.58)									
**6.5.2 <1month**									
Rutten2019	-28.4	2.3	35	-28.3	2.7	33	43.1%	-0.04 [-0.52, 0.44]	2019
Shen2022	-22.17	4.44	23	-22.91	3.84	23	29.1%	0.18 [-0.40, 0.75]	2022
**Subtotal (95% CI)**			**58**			**56**	**72.2%**	**0.05 [-0.32, 0.41]**	
Heterogeneity: Chi² = 0.32, df = 1 (P = 0.57); I² = 0%									
Test for overall effect: Z = 0.25 (P = 0.80)									
**Total (95% CI)**			**80**			**78**	**100.0%**	**0.08 [-0.23, 0.39]**	
Heterogeneity: Chi² = 0.43, df = 2 (P = 0.81); I² = 0%									
Test for overall effect: Z = 0.51 (P = 0.61)									
Test for subgroup differences: Chi² = 0.12, df = 1 (P = 0.73), I² = 0%									

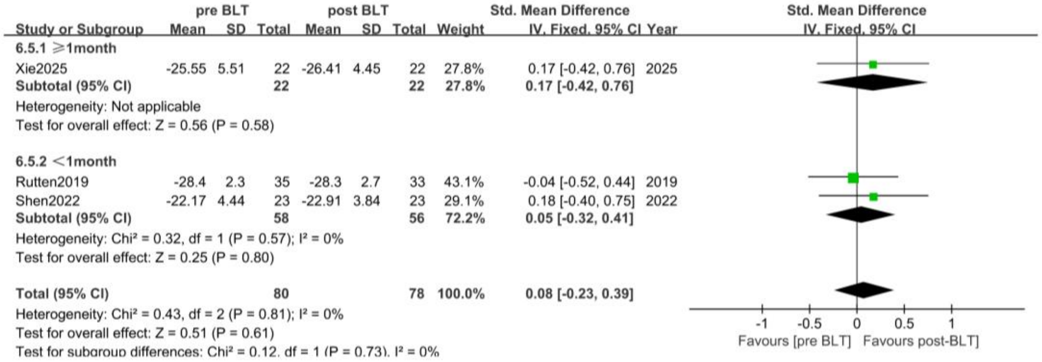

(c)Cognition

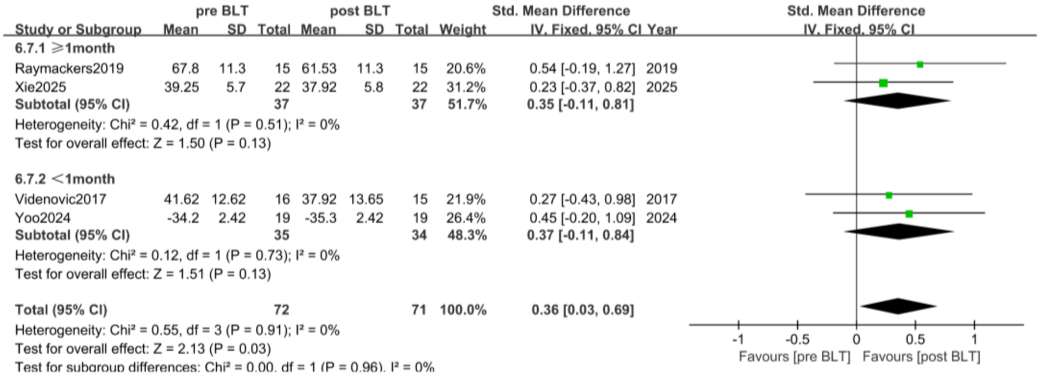

(d)Fatigue

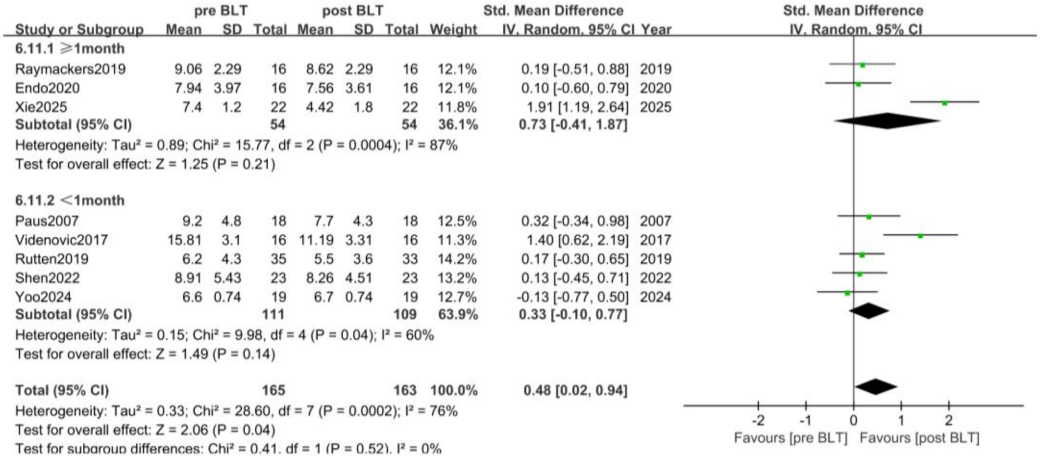

(e)Daytime sleepiness

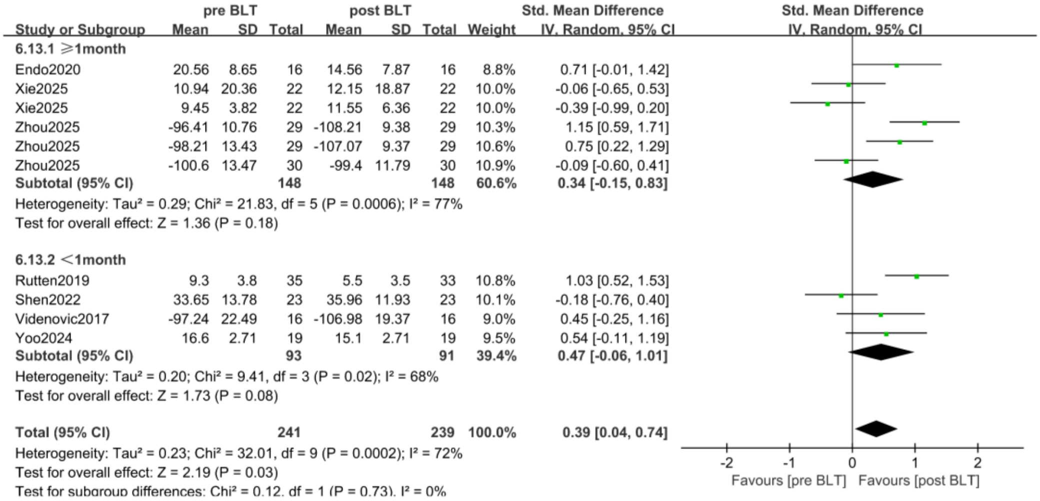

(f)Nighttime sleep

Study or Subgroup	pre BLT Mean	pre BLT SD	pre BLT Total	post BLT Mean	post BLT SD	post BLT Total	Weight	Std. Mean Difference IV, Random, 95% CI	Year
**6.16.1 ≥1month**									
Rutten2019	4.3	1.5	35	2.3	1.5	33	27.2%	1.32 [0.79, 1.85]	2019
Xie2025	8.83	5.73	22	7	3.82	22	25.1%	0.37 [-0.23, 0.97]	2025
**Subtotal (95% CI)**			**57**			**55**	**52.3%**	**0.85 [-0.08, 1.78]**	
Heterogeneity: Tau² = 0.37; Chi² = 5.46, df = 1 (P = 0.02); I² = 82%									
Test for overall effect: Z = 1.80 (P = 0.07)									
**6.16.2 <1month**									
Videnovic2017	7.88	4.11	16	6.25	4.27	16	22.2%	0.38 [-0.32, 1.08]	2017
Shen2022	9.22	4.74	23	7.65	3.79	23	25.5%	0.36 [-0.22, 0.94]	2022
**Subtotal (95% CI)**			**39**			**39**	**47.7%**	**0.37 [-0.08, 0.82]**	
Heterogeneity: Tau² = 0.00; Chi² = 0.00, df = 1 (P = 0.97); I² = 0%									
Test for overall effect: Z = 1.61 (P = 0.11)									
**Total (95% CI)**			**96**			**94**	**100.0%**	**0.63 [0.12, 1.13]**	
Heterogeneity: Tau² = 0.17; Chi² = 8.53, df = 3 (P = 0.04); I² = 65%									
Test for overall effect: Z = 2.44 (P = 0.01)									
Test for subgroup differences: Chi² = 0.85, df = 1 (P = 0.36), I² = 0%									

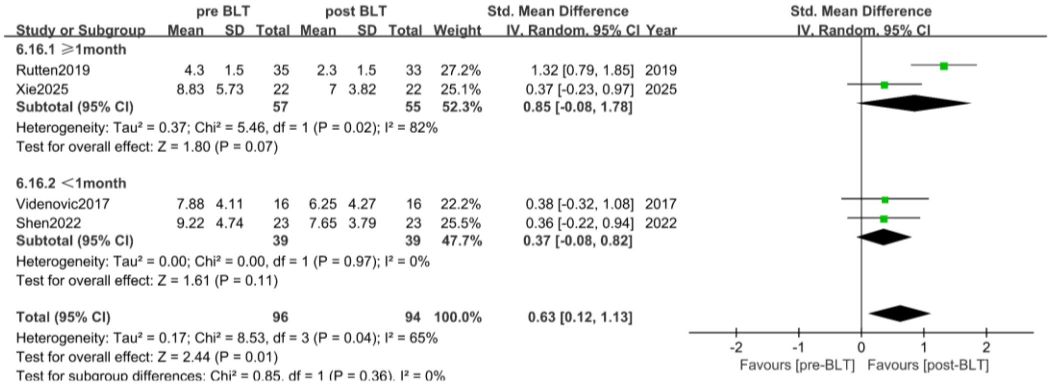

(g)Sleep quality

Study or Subgroup	pre BLT Mean	pre BLT SD	pre BLT Total	post BLT Mean	post BLT SD	post BLT Total	Weight	Std. Mean Difference IV, Fixed, 95% CI
**6.9.1 ≥1month**								
Xie2025	8	3.39	22	8.14	6.15	22	17.0%	-0.03 [-0.62, 0.56]
Xie2025	13.9	16.75	22	8.76	13.15	22	16.8%	0.34 [-0.26, 0.93]
**Subtotal (95% CI)**			**44**			**44**	**33.8%**	**0.15 [-0.27, 0.57]**
Heterogeneity: Chi² = 0.72, df = 1 (P = 0.40); I² = 0%								
Test for overall effect: Z = 0.71 (P = 0.48)								
**6.9.2 <1month**								
Paus2007	12.6	6.8	18	11	7.7	18	13.9%	0.22 [-0.44, 0.87]
Rutten2019	-79	13.1	35	-83.3	13.6	33	26.0%	0.32 [-0.16, 0.80]
Videnovic2017	41.46	19.3	16	43	14.84	16	12.4%	-0.09 [-0.78, 0.61]
Willis2018	4.12	3.5	9	3.93	4.81	9	7.0%	0.04 [-0.88, 0.97]
Willis2018	0.19	0.44	9	0.19	0.44	9	7.0%	0.00 [-0.92, 0.92]
**Subtotal (95% CI)**			**87**			**85**	**66.2%**	**0.16 [-0.14, 0.46]**
Heterogeneity: Chi² = 1.11, df = 4 (P = 0.89); I² = 0%								
Test for overall effect: Z = 1.03 (P = 0.30)								
**Total (95% CI)**			**131**			**129**	**100.0%**	**0.16 [-0.09, 0.40]**
Heterogeneity: Chi² = 1.83, df = 6 (P = 0.93); I² = 0%								
Test for overall effect: Z = 1.26 (P = 0.21)								
Test for subgroup differences: Chi² = 0.00, df = 1 (P = 0.98), I² = 0%								

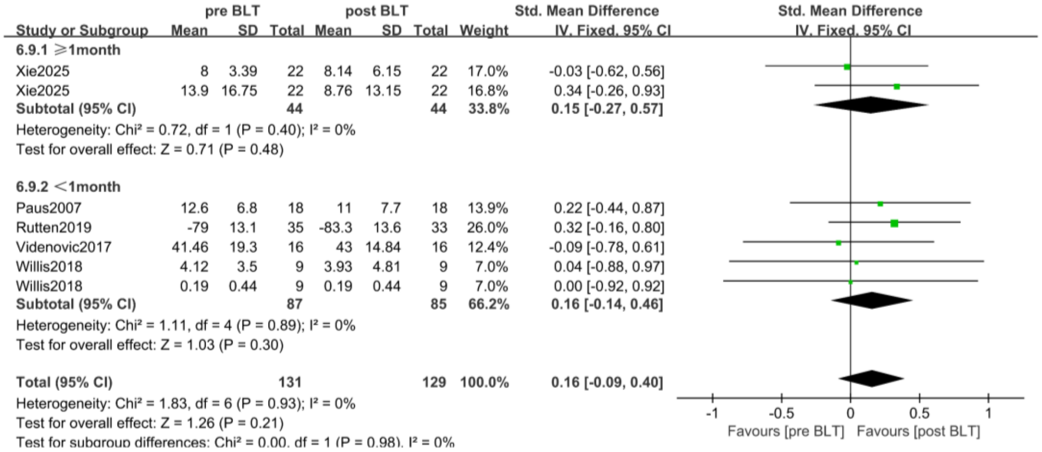

(h)Quality of life

Supplementary Figure10 The BLT cycle affects the effect

Supplementary Figure11

Study or Subgroup	pre-BLT Mean	pre-BLT SD	pre-BLT Total	post-BLT Mean	post-BLT SD	post-BLT Total	Weight	Mean Difference IV, Random, 95% CI	Year
**7.2.1 >50cm**									
Videnovic2017	8.31	3.63	16	7.88	4.53	16	13.8%	0.43 [-2.41, 3.27]	2017
Willis2018	8	13.99	9	6.97	11.37	9	3.0%	1.03 [-10.75, 12.81]	2018
Shen2022	7.96	4.25	23	8.52	4.03	23	14.8%	-0.56 [-2.95, 1.83]	2022
Xie2025	5.84	8.06	22	5.79	7.21	22	10.3%	0.05 [-4.47, 4.57]	2025
**Subtotal (95% CI)**			**70**			**70**	**41.8%**	**-0.10 [-1.78, 1.58]**	
Heterogeneity: Tau² = 0.00; Chi² = 0.31, df = 3 (P = 0.96); I² = 0%									
Test for overall effect: Z = 0.11 (P = 0.91)									
**7.2.2 ≤50cm**									
Paus2007	9.9	6.1	18	7.7	5.1	18	12.0%	2.20 [-1.47, 5.87]	2007
Rutten2019	14.7	3.5	35	7.6	5	33	15.5%	7.10 [5.04, 9.16]	2019
Rutten2019	17.9	5.9	35	13.7	6.9	33	13.3%	4.20 [1.14, 7.26]	2019
Yoo2024	10.6	1.56	20	10	1.56	20	17.3%	0.60 [-0.37, 1.57]	2024
**Subtotal (95% CI)**			**108**			**104**	**58.2%**	**3.51 [-0.11, 7.13]**	
Heterogeneity: Tau² = 11.92; Chi² = 33.53, df = 3 (P < 0.00001); I² = 91%									
Test for overall effect: Z = 1.90 (P = 0.06)									
**Total (95% CI)**			**178**			**174**	**100.0%**	**2.04 [-0.17, 4.25]**	
Heterogeneity: Tau² = 7.12; Chi² = 38.53, df = 7 (P < 0.00001); I² = 82%									
Test for overall effect: Z = 1.81 (P = 0.07)									
Test for subgroup differences: Chi² = 3.14, df = 1 (P = 0.08), I² = 68.2%									

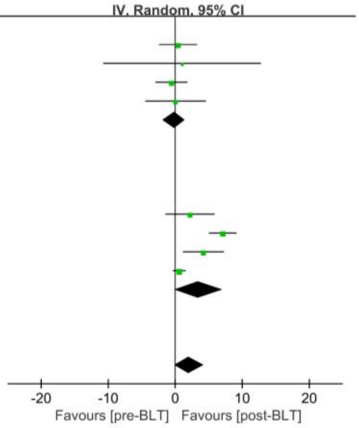

(a)Depression

Study or Subgroup	pre-BLT Mean	pre-BLT SD	pre-BLT Total	post-BLT Mean	post-BLT SD	post-BLT Total	Weight	Mean Difference IV, Fixed, 95% CI	Year
**7.3.1 >50cm**									
Xie2025	5.82	6.36	22	4.46	6.15	20	6.6%	1.36 [-2.43, 5.15]	2025
**Subtotal (95% CI)**			**22**			**20**	**6.6%**	**1.36 [-2.43, 5.15]**	
Heterogeneity: Not applicable									
Test for overall effect: Z = 0.70 (P = 0.48)									
**7.3.2 ≤50cm**									
Yoo2024	10.6	1.62	20	10	1.62	20	93.4%	0.60 [-0.40, 1.60]	2024
**Subtotal (95% CI)**			**20**			**20**	**93.4%**	**0.60 [-0.40, 1.60]**	
Heterogeneity: Not applicable									
Test for overall effect: Z = 1.17 (P = 0.24)									
**Total (95% CI)**			**42**			**40**	**100.0%**	**0.65 [-0.32, 1.62]**	
Heterogeneity: Chi² = 0.14, df = 1 (P = 0.70); I² = 0%									
Test for overall effect: Z = 1.31 (P = 0.19)									
Test for subgroup differences: Chi² = 0.14, df = 1 (P = 0.70), I² = 0%									

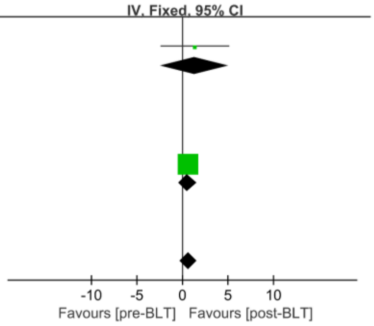

(b)Anxiety

Study or Subgroup	pre-BLT Mean	pre-BLT SD	pre-BLT Total	post-BLT Mean	post-BLT SD	post-BLT Total	Weight	Mean Difference IV, Fixed, 95% CI	Year
**7.5.1 >50cm**									
Shen2022	-22.17	4.44	23	-22.91	3.84	23	17.6%	0.74 [-1.66, 3.14]	2022
Xie2025	-25.55	5.51	22	-26.41	4.45	22	11.6%	0.86 [-2.10, 3.82]	2025
**Subtotal (95% CI)**			**45**			**45**	**29.2%**	**0.79 [-1.08, 2.65]**	
Heterogeneity: Chi² = 0.00, df = 1 (P = 0.95); I² = 0%									
Test for overall effect: Z = 0.83 (P = 0.41)									
**7.5.2 ≤50cm**									
Rutten2019	-28.4	2.3	35	-28.3	2.7	33	70.8%	-0.10 [-1.30, 1.10]	2019
**Subtotal (95% CI)**			**35**			**33**	**70.8%**	**-0.10 [-1.30, 1.10]**	
Heterogeneity: Not applicable									
Test for overall effect: Z = 0.16 (P = 0.87)									
**Total (95% CI)**			**80**			**78**	**100.0%**	**0.16 [-0.85, 1.17]**	
Heterogeneity: Chi² = 0.62, df = 2 (P = 0.73); I² = 0%									
Test for overall effect: Z = 0.31 (P = 0.76)									
Test for subgroup differences: Chi² = 0.62, df = 1 (P = 0.43), I² = 0%									

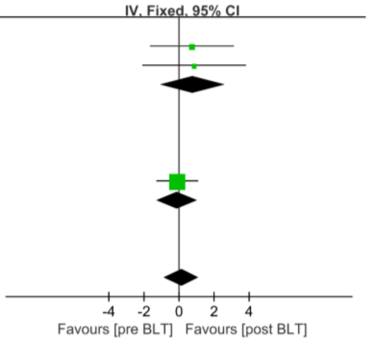

(c)Cognition

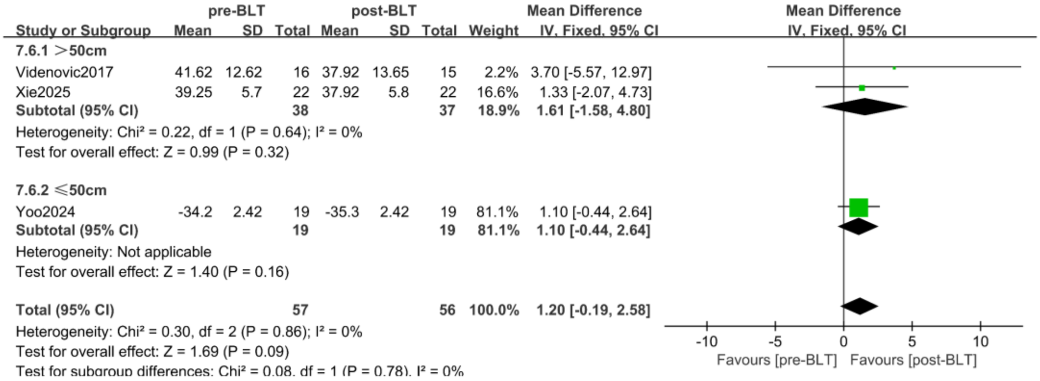

(d)Fatigue

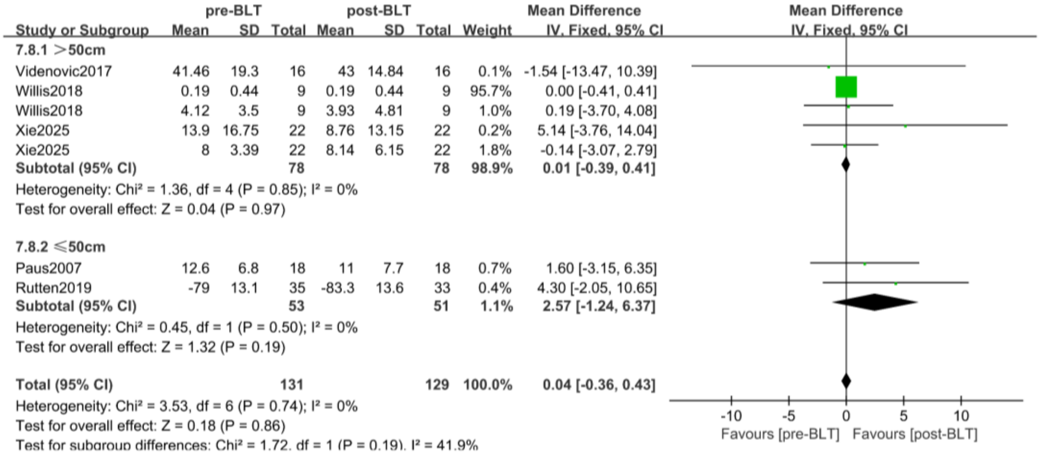

(e) Quality of life

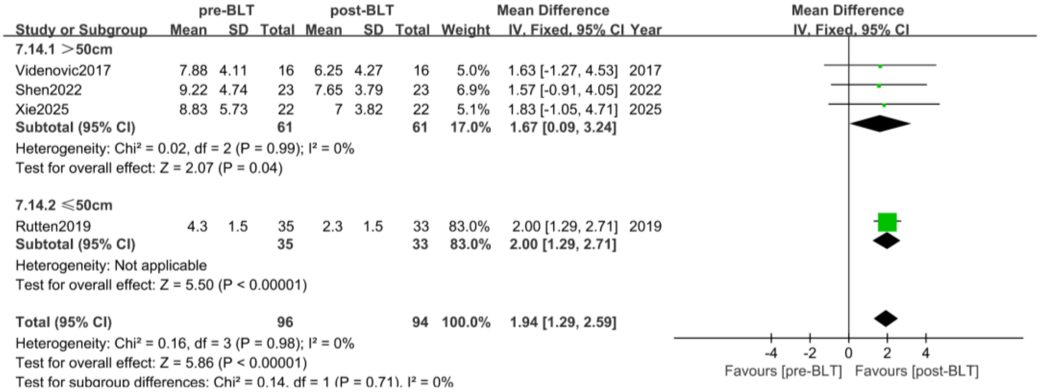

(f)Sleep quality

Supplementary Figure11 The influence of light source distance on the therapeutic effect of BLT

Supplementary Figure12

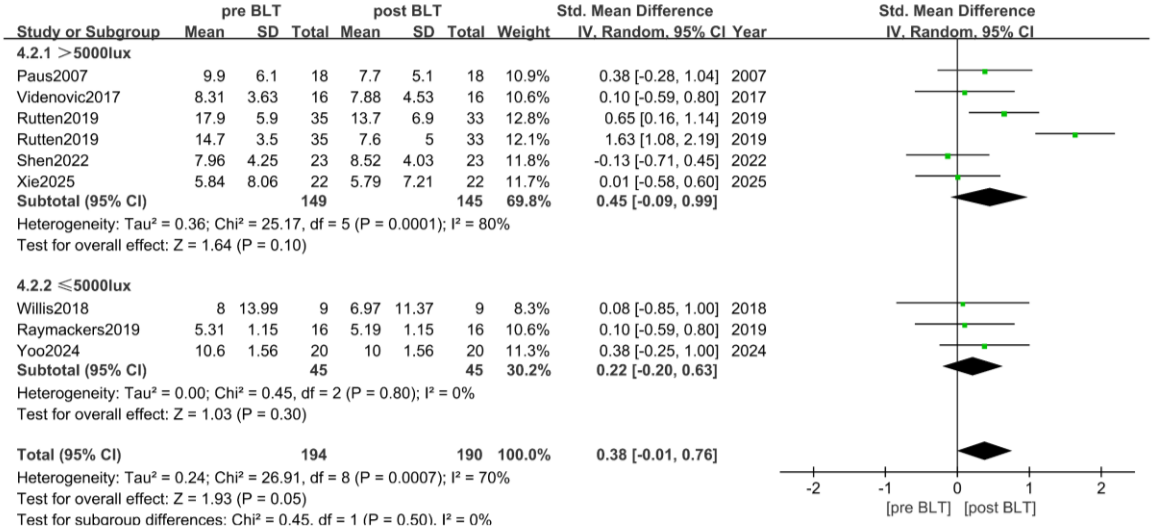

(a)Depression

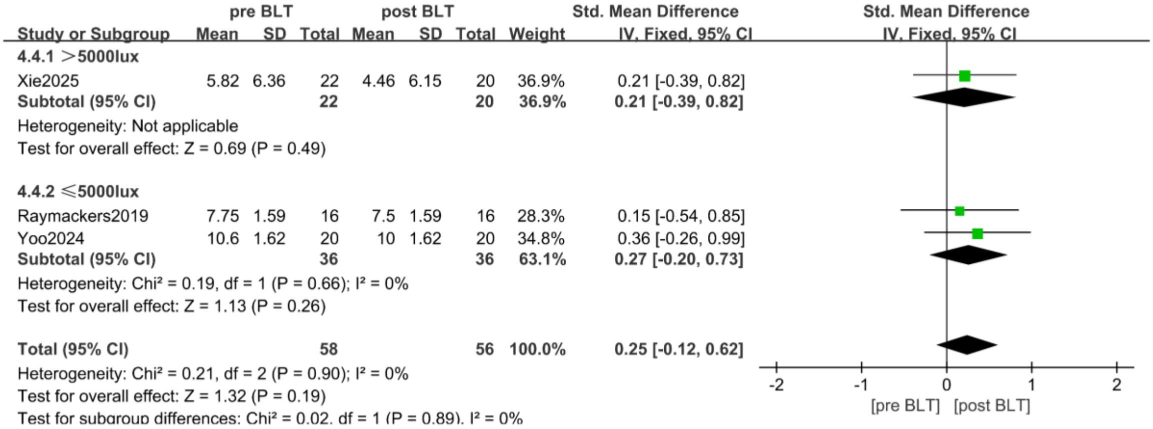

(b)Anxiety

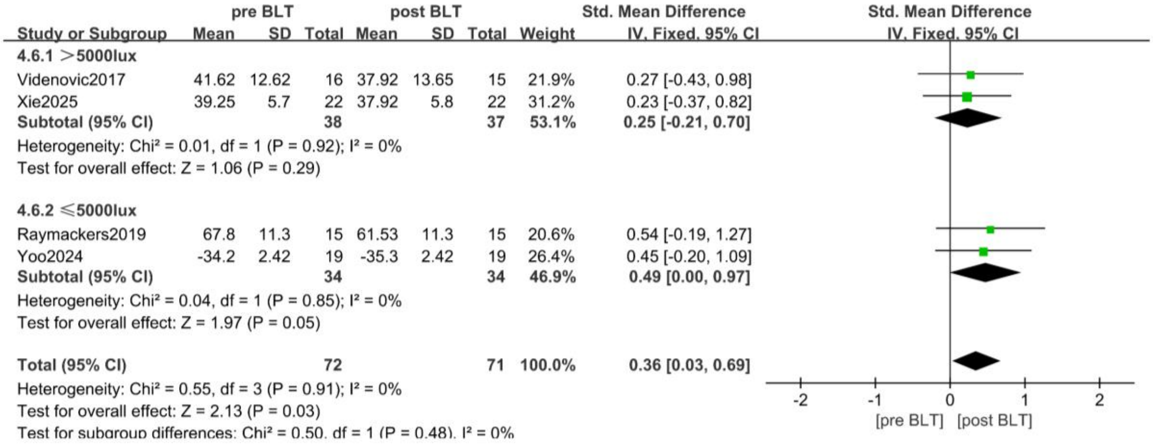

(c)Fatigue

Study or Subgroup	pre BLT Mean	pre BLT SD	pre BLT Total	post BLT Mean	post BLT SD	post BLT Total	Weight	Std. Mean Difference IV, Random, 95% CI
**4.10.1 ＞5000lux**								
Paus2007	9.2	4.8	18	7.7	4.3	18	12.5%	0.32 [-0.34, 0.98]
Rutten2019	6.2	4.3	35	5.5	3.6	33	14.2%	0.17 [-0.30, 0.65]
Shen2022	8.91	5.43	23	8.26	4.51	23	13.2%	0.13 [-0.45, 0.71]
Videnovic2017	15.81	3.1	16	11.19	3.31	16	11.3%	1.40 [0.62, 2.19]
Xie2025	7.4	1.2	22	4.42	1.8	22	11.8%	1.91 [1.19, 2.64]
**Subtotal (95% CI)**			**114**			**112**	**63.0%**	**0.75 [0.08, 1.43]**
Heterogeneity: Tau² = 0.48; Chi² = 22.97, df = 4 (P = 0.0001); I² = 83%								
Test for overall effect: Z = 2.19 (P = 0.03)								
**4.10.3 ≤5000lux**								
Endo2020	7.94	3.97	16	7.56	3.61	16	12.1%	0.10 [-0.60, 0.79]
Raymackers2019	9.06	2.29	16	8.62	2.29	16	12.1%	0.19 [-0.51, 0.88]
Yoo2024	6.6	0.74	19	6.7	0.74	19	12.7%	-0.13 [-0.77, 0.50]
**Subtotal (95% CI)**			**51**			**51**	**37.0%**	**0.04 [-0.35, 0.43]**
Heterogeneity: Tau² = 0.00; Chi² = 0.48, df = 2 (P = 0.79); I² = 0%								
Test for overall effect: Z = 0.20 (P = 0.84)								
**Total (95% CI)**			**165**			**163**	**100.0%**	**0.48 [0.02, 0.94]**
Heterogeneity: Tau² = 0.33; Chi² = 28.60, df = 7 (P = 0.0002); I² = 76%								
Test for overall effect: Z = 2.06 (P = 0.04)								
Test for subgroup differences: Chi² = 3.22, df = 1 (P = 0.07), I² = 68.9%								

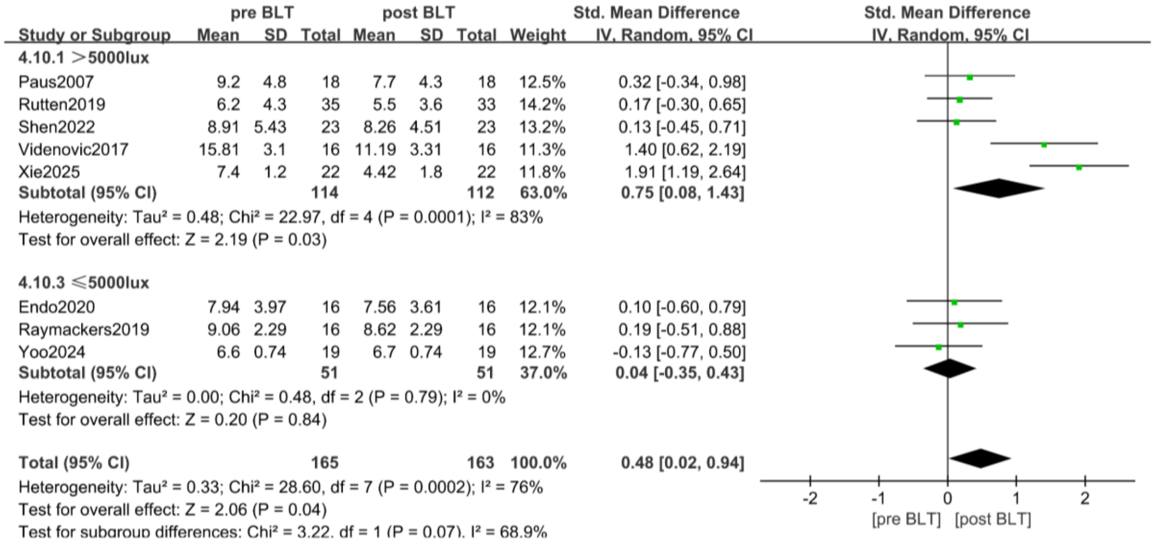

(d)Daytime sleepiness

Study or Subgroup	pre BLT Mean	pre BLT SD	pre BLT Total	post BLT Mean	post BLT SD	post BLT Total	Weight	Std. Mean Difference IV, Random, 95% CI	Year
**5.12.1 ＞5000lux**									
Videnovic2017	-97.24	22.49	16	-106.98	19.37	16	9.0%	0.45 [-0.25, 1.16]	2017
Rutten2019	9.3	3.8	35	5.5	3.5	35	10.9%	1.03 [0.53, 1.53]	2019
Shen2022	33.65	13.78	23	35.96	11.93	23	10.1%	-0.18 [-0.76, 0.40]	2022
Xie2025	10.94	20.36	22	12.15	18.87	22	10.0%	-0.06 [-0.65, 0.53]	2025
Xie2025	9.45	3.82	22	11.55	6.36	22	10.0%	-0.39 [-0.99, 0.20]	2025
Zhou2025	-96.41	10.76	29	-108.21	9.38	29	10.3%	1.15 [0.59, 1.71]	2025
**Subtotal (95% CI)**			**147**			**147**	**60.3%**	**0.34 [-0.20, 0.88]**	
Heterogeneity: Tau² = 0.37; Chi² = 25.97, df = 5 (P < 0.0001); I² = 81%									
Test for overall effect: Z = 1.24 (P = 0.22)									
**5.12.2 ≤5000lux**									
Endo2020	20.56	8.65	16	14.56	7.87	16	8.8%	0.71 [-0.01, 1.42]	2020
Yoo2024	16.6	2.71	19	15.1	2.71	19	9.5%	0.54 [-0.11, 1.19]	2024
Zhou2025	-100.6	13.47	30	-99.4	11.79	30	10.8%	-0.09 [-0.60, 0.41]	2025
Zhou2025	-98.21	13.43	29	-107.07	9.37	29	10.6%	0.75 [0.22, 1.29]	2025
**Subtotal (95% CI)**			**94**			**94**	**39.7%**	**0.45 [0.02, 0.88]**	
Heterogeneity: Tau² = 0.10; Chi² = 6.21, df = 3 (P = 0.10); I² = 52%									
Test for overall effect: Z = 2.07 (P = 0.04)									
**Total (95% CI)**			**241**			**241**	**100.0%**	**0.39 [0.04, 0.74]**	
Heterogeneity: Tau² = 0.23; Chi² = 32.22, df = 9 (P = 0.0002); I² = 72%									
Test for overall effect: Z = 2.19 (P = 0.03)									
Test for subgroup differences: Chi² = 0.10, df = 1 (P = 0.76), I² = 0%									

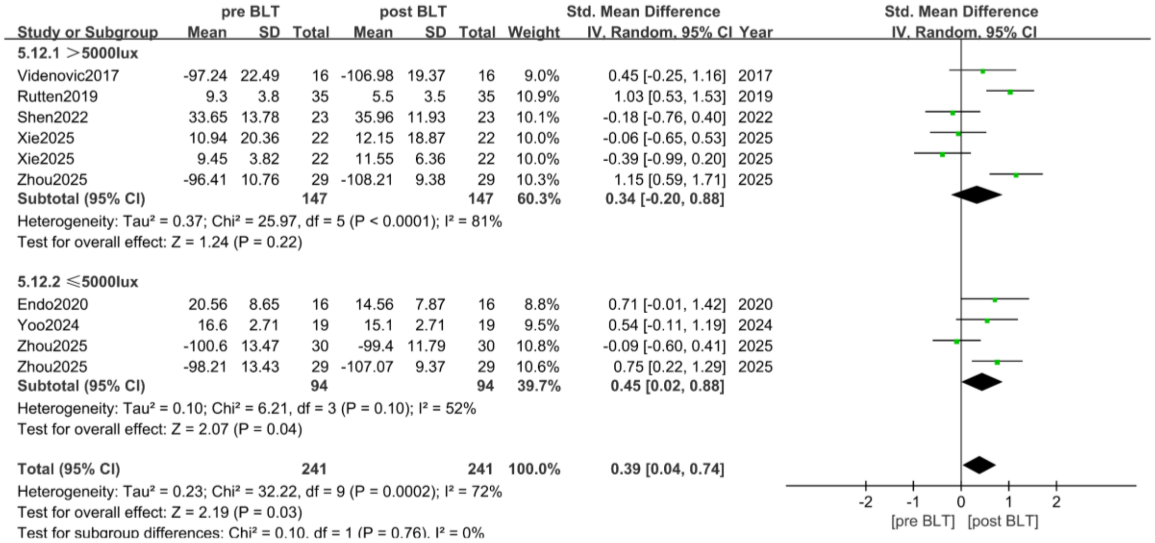

(e)Nighttime sleep

Study or Subgroup	pre BLT Mean	pre BLT SD	pre BLT Total	post BLT Mean	post BLT SD	post BLT Total	Weight	Std. Mean Difference IV, Fixed, 95% CI
**5.8.1 ＞5000lux**								
Paus2007	12.6	6.8	18	11	7.7	18	13.9%	0.22 [-0.44, 0.87]
Rutten2019	-79	13.1	35	-83.3	13.6	33	26.0%	0.32 [-0.16, 0.80]
Videnovic2017	41.46	19.3	16	43	14.84	16	12.4%	-0.09 [-0.78, 0.61]
Xie2025	13.9	16.75	22	8.76	13.15	22	16.8%	0.34 [-0.26, 0.93]
Xie2025	8	3.39	22	8.14	6.15	22	17.0%	-0.03 [-0.62, 0.56]
**Subtotal (95% CI)**			**113**			**111**	**86.1%**	**0.18 [-0.08, 0.44]**
Heterogeneity: $Chi^2 = 1.64, df = 4 (P = 0.80); I^2 = 0\%$								
Test for overall effect: Z = 1.33 (P = 0.18)								
**5.8.2 ≤5000lux**								
Willis2018	0.19	0.44	9	0.19	0.44	9	7.0%	0.00 [-0.92, 0.92]
Willis2018	4.12	3.5	9	3.93	4.81	9	7.0%	0.04 [-0.88, 0.97]
**Subtotal (95% CI)**			**18**			**18**	**13.9%**	**0.02 [-0.63, 0.67]**
Heterogeneity: $Chi^2 = 0.00, df = 1 (P = 0.95); I^2 = 0\%$								
Test for overall effect: Z = 0.06 (P = 0.95)								
**Total (95% CI)**			**131**			**129**	**100.0%**	**0.16 [-0.09, 0.40]**
Heterogeneity: $Chi^2 = 1.83, df = 6 (P = 0.93); I^2 = 0\%$								
Test for overall effect: Z = 1.26 (P = 0.21)								
Test for subgroup differences: $Chi^2 = 0.19, df = 1 (P = 0.66), I^2 = 0\%$								

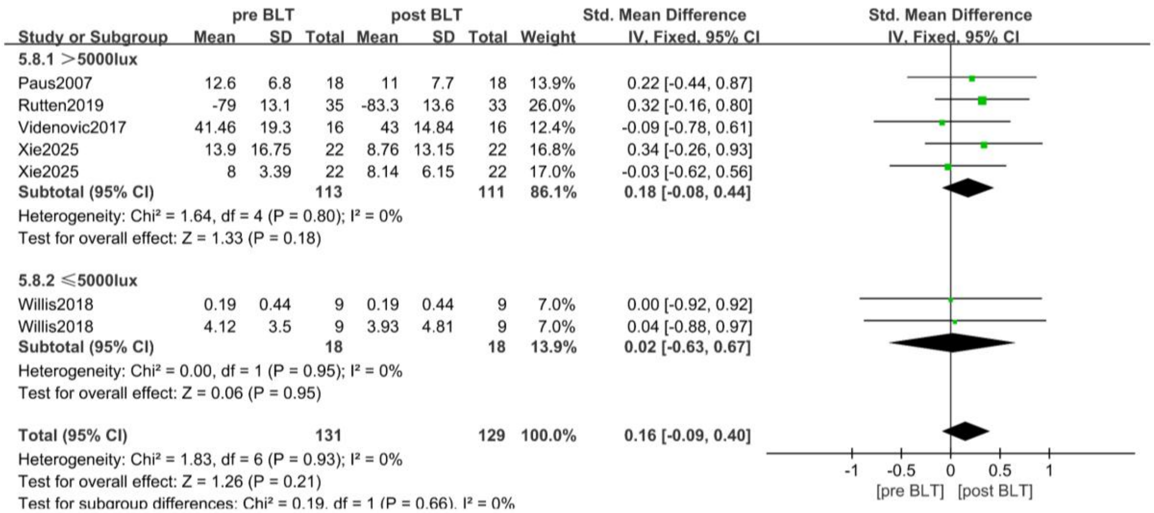

(f)Quality of life

Supplementary Figure12 The intensity of BLT affects the effect
